# A multi-parameter diagnostic clinical decision tree for the rapid diagnosis of tuberculosis in HIV-positive patients presenting to an emergency centre

**DOI:** 10.12688/wellcomeopenres.15824.1

**Published:** 2020-04-17

**Authors:** Daniël Jacobus van Hoving, Graeme Meintjes, Gary Maartens, Andre Pascal Kengne

**Affiliations:** 1 Division of Emergency Medicine, University of Cape Town, Cape Town, Western Cape, 7935, South Africa; 2 Division of Emergency Medicine, Stellenbosch University, Cape Town, Western Cape, 7505, South Africa; 3 Wellcome Centre for Infectious Diseases Research in Africa, Institute of Infectious Disease and Molecular Medicine, University of Cape Town, Cape Town, Western Cape, 7935, South Africa; 4 Department of Medicine, University of Cape Town, Cape Town, Western Cape, 7935, South Africa; 5 Division of Clinical Pharmacology, Department of Medicine, University of Cape Town, Cape Town, Western Cape, 7935, South Africa; 6 Non-Communicable Diseases Research Unit, South African Medical Research Council, Cape Town, Western Cape, 7505, South Africa

**Keywords:** HIV, tuberculosis, algorithm, emergency, lipoarabinomannan, point-of-care, ultrasound, X-ray

## Abstract

**Background:** Early diagnosis is essential to reduce the morbidity and mortality of HIV-associated tuberculosis. We developed a multi-parameter clinical decision tree to facilitate rapid diagnosis of tuberculosis using point-of-care diagnostic tests in HIV-positive patients presenting to an emergency centre.

**Methods: **A cross-sectional study was performed in a district hospital emergency centre in a high-HIV-prevalence community in South Africa. Consecutive HIV-positive adults with ≥1 WHO tuberculosis symptoms were enrolled over a 16-month period. Point-of-care ultrasound (PoCUS) and urine lateral flow lipoarabinomannan (LF-LAM) assay were done according to standardized protocols. Participants also received a chest X-ray. Reference standard was the detection of *Mycobacterium tuberculosis* using Xpert MTB/RIF or culture. Logistic regressions models were used to investigate the independent association between prevalent microbiologically confirmed tuberculosis and clinical and biological variables of interest. A decision tree model to predict tuberculosis was developed using the classification and regression tree algorithm.

**Results: **There were 414 participants enrolled: 171 male, median age 36 years, median CD4 cell count 86 cells/mm ^3^. Tuberculosis prevalence was 42% (n=172). Significant variables used to build the classification tree included ≥2 WHO symptoms, antiretroviral therapy use, LF-LAM, PoCUS independent features (pericardial effusion, ascites, intra-abdominal lymphadenopathy) and chest X-ray. LF-LAM was positioned after WHO symptoms (75% true positive rate, representing 17% of study population). Chest X-ray should be performed next if LF-LAM is negative. The presence of ≤1 PoCUS independent feature in those with ‘possible or unlikely tuberculosis’ on chest x-ray represented 47% of non-tuberculosis participants (true negative rate 83%). In a prediction tree which only included true point-of-care tests, a negative LF-LAM and the presence of ≤2 independent PoCUS features had a 71% true negative rate (representing 53% of sample).

**Conclusions:** LF-LAM should be performed in all adults with suspected HIV-associated tuberculosis (regardless of CD4 cell count) presenting to the emergency centre.

## Introduction

Tuberculosis remains an important cause of morbidity and mortality globally, despite ongoing control efforts ^1^. The early diagnosis and successful treatment of people with tuberculosis should reduce the risk of mortality and morbidity, and decrease the transmission of tuberculosis ^2^. Factors associated with delays in the diagnosis of tuberculosis include the limitations of tuberculosis diagnostic tests, limited availability of these tests in high burden settings, and the reduced diagnostic performance of tuberculosis tests in people living with HIV (PLWH) ^3– 5^. In PLWH with advanced immunosuppression, the diagnosis of active tuberculosis is challenging due to more atypical clinical presentations; other opportunistic infections with similar presentations; high proportion with inability to produce sputum or negative sputum smears; and high rates of extra-pulmonary and disseminated tuberculosis ^6– 11^. Autopsy studies in HIV-positive adults report a very high proportion with tuberculosis (32% to 47%), almost half (46%) of which was undiagnosed pre-mortem ^12^.

The WHO recommends that HIV-positive patients should be systematically screened for active tuberculosis when visiting a healthcare facility ^2^. Many patients access the healthcare system through hospital emergency centres. The prevalence of HIV-related admissions to emergency centres varies, with up to 43% documented in Uganda ^13^. These patients are often severely ill and would benefit from prompt diagnosis and treatment of tuberculosis to decrease mortality ^14^.

The use of point-of-care diagnostic tests would facilitate rapid diagnosis of tuberculosis. Lateral flow lipoarabinomannan (LF-LAM) is currently the only true point-of-care test, with other tests (e.g. smear microscopy, Xpert MTB/RIF, Xpert MTB/RIF Ultra, GeneXpert OMNI, and portable digital chest X-ray) being near point-of-care tests ^15^. Point-of-care ultrasound (PoCUS) is also a potentially useful test for extra-pulmonary or disseminated tuberculosis ^16^. No evidence-based algorithm incorporating clinical information, individual PoCUS features, and urine LF-LAM for diagnosing tuberculosis in HIV-positive patients currently exists. We performed a cross-sectional diagnostic study and developed a multi-parameter clinical decision tree to facilitate rapid diagnosis of tuberculosis in HIV-positive patients presenting to an emergency centre.

## Methods

### Study setting and participants

Khayelitsha is a township with a mix of formal and informal housing in Cape Town, South Africa. The Khayelitsha Health sub-district has an antenatal HIV prevalence of 34% ^17^, and an annual tuberculosis notification rate of 917 per 100,000 persons ^18^. The emergency centre of Khayelitsha Hospital (a district-level hospital) manages ± 35,000 patients per annum with an admission rate around 30%. The HIV prevalence of patients managed in the resuscitation unit is 23% ^19^.

Inclusion criteria were adults (≥18 years); HIV-positive (HIV-status was determined by laboratory confirmation or from the clinical records), and presence of at least one symptom of the WHO's recommended four-symptom screening rule for tuberculosis in PLWH (cough of any duration, fever, drenching night sweats, or weight loss) ^20^. Exclusion criteria were: presenting to the emergency centre more than 24 hours before screening; received anti-tuberculosis treatment within 3 months of screening; pregnant; main clinical presentation of meningitis syndrome or new focal neurology; trauma, gynaecological or psychiatric presentation. Data from this cohort relating to LF-LAM and PoCUS were previously published ^21, 22^. These manuscripts described the use of LF-LAM in an acute care setting and identified PoCUS features independently associated with HIV-associated tuberculosis ^21, 22^.

All participants provided written informed consent using a two-phase consent process. Severely ill participants were provided with a short one-page consent form indicating what extra tests would be done and that these would be used to facilitate diagnosis of tuberculosis and for research purposes. Full consent was obtained once patients had recovered and agreed to participate. The study was approved by the Human Research Ethics Committee of the University of Cape Town (HREC REF: 697/2015).

### Procedures and samples

Consecutive patients evaluated at the emergency centre were screened for eligibility from June 2016 through October 2017. A standardized data collection form was used to record demographic and clinical information. Urine, sputum and blood samples were obtained from all patients whenever possible (see *Extended data*) ^23^. Fresh urine samples were tested using the Xpert MTB/RIF assay (GX4) (Cepheid Inc., Sunnyvale, CA, USA) and for the presence of LAM (Alere Determine™ TB LAM Ag test, Alere Inc., Waltham, MA, USA); LF-LAM was performed in the emergency centre ^21^. Sputum specimens were tested using the Xpert MTB/RIF assay (GX4) and cultured in mycobacterial growth indicator tubes (MGIT; Becton Dickson, Sparks, MD, USA). Mycobacterial blood cultures were performed using the BACTEC MYCO/F Lytic blood culture bottle (Becton Dickson, Sparks, MD, USA). The MTBDR *plus* assay (Hain Lifescience, Nehren, Germany) were used to identify culture isolates as *M. tuberculosis* complex. Complete blood count and CD4 cell count were done as part of routine clinical care. CD4 cell count results were accepted if performed within 3 months of enrolment. The National Health Laboratory Service performed all the tests.

Ultrasound examination was performed in the emergency centre and the findings documented on a standardized assessment form. A single, emergency physician (with adequate training and credentials as specified by the International Federation of Emergency Medicine’s Emergency Ultrasound Special Interest Group ^24^) performed the ultrasound examination using either a Mindray M5™ ultrasound system with a 3C5s (2.5–6.5 MHz) convex probe and a 7L4s (5.0–10 MHz) linear probe (Mindray DS USA, Inc., Mahwah, NJ, USA) or a NanoMaxx™ ultrasound system with a L38n (10-5 MHz) linear array probe and a C60n (5–2 MHz) curved array probe (SonoSite Inc., Bothell, WA, USA). Ultrasound examinations were performed before any specimens were collected. At the time of the ultrasound, the point-of-care sonographer had access to the clinical information but not to results from the reference standard (detection of *M. tuberculosis* from Xpert MTB/RIF and/or culture on any specimen obtained from any anatomical site).

Chest x-rays were reviewed by a single radiologist using a standardized assessment form (see *Extended dat*a ^23^. Chest x-rays were classified as unlikely tuberculosis, probable tuberculosis, and likely tuberculosis. The radiologist had no access to clinical information or the reference standard.

### Statistical analyses

The sample size was determined with the aim of including more than the recommended 10 candidate predictors (including interaction terms) from multivariable logistic regression analyses ^25^. The tuberculosis prevalence in HIV-positive patients in the emergency centre is around 25%, ^19^ and a sample size of 400 HIV-positive participants was deemed adequate to include 100 tuberculosis cases. Data were analysed with the use of SAS/STAT ^®^ software (Version 9.4 of the SAS System for Windows [Copyright © 2019 SAS Institute Inc. SAS and all other SAS Institute Inc. product or service names are registered trademarks or trademarks of SAS Institute Inc., Cary, NC, USA]), R statistical software version 3.4.3 (2017-11-30) [The R® Foundation for Statistical Computing Platform], and SPSS Statistics for Windows, Version 25.0 (IBM Corp. Released 2017. Armonk, NY: IBM Corp.). Group comparisons used χ2 test and variants for qualitative variables and Student’s t-test or non-parametric equivalents for continuous variables. Results are presented as count (percentages), mean and standard deviation (SD) or median and 25th-75th percentiles as appropriate.

Logistic regressions models were used to investigate the independent association between prevalent microbiologically confirmed tuberculosis and clinical and biological variables of interest. Candidate variables included WHO symptom screen (presence of cough, ≥1 present, ≥2 present, ≥3 present), antiretroviral therapy status (currently on antiretroviral medicine), presence and number of WHO danger signs (≥1 present, ≥2 present, ≥3 present) ^26^, number of individual PoCUS features (≥1 present, ≥2 present, ≥3 present), number of PoCUS features independently associated with tuberculosis (≥1 present, ≥2 present, ≥3 present), urinary LF-LAM, haemoglobin, chest X-ray (possible tuberculosis, likely tuberculosis, possible and likely tuberculosis) and CD4 cell count (<100 cells/mm ^3^, 100–200 cells/mm ^3^, >200 cells/mm ^3^). Individual PoCUS features included any sized pericardial effusion, pleural effusion, ascites, any focal splenic lesion, and any sized intra-abdominal lymphadenopathy. PoCUS features independently associated with tuberculosis (pericardial effusion of any size, ascites, intra-abdominal lymphadenopathy of any size) were determined by multivariable logistical regression ^22^.

For correlated variables, when more than one index was significant in a univariate model, the one with more significant effect on the -2log $\hat{L}$ statistic was first entered into the multivariable model. However, in the final model, the effect of substituting variables was also assessed. When more than one correlated variable was significant in multivariable models, the final model selected was the one associated with the smallest Akaike’s information criterion (AIC), a statistic derived from the -2log $\hat{L}$ statistic. Multivariable model building was based on the combination of significant variables in univariable models (based on a threshold p<0.10). A model comprising WHO screening symptoms and history of current antiretroviral therapy use was used as starting model ^20^. The ability of logistic regression models to discriminate between participants who had and those who did not have microbiologically confirmed tuberculosis was assessed using area under the receiver operating characteristic curves (AUC) and the relative integrated discrimination improvement (RIDI) which measures the percentage increase in discrimination when an extra variable is added to a prediction model ^27, 28^. AUC comparisons used nonparametric methods ^29^. Bootstrap techniques were used to derive the 95% confidence interval (CI) for the RIDI estimates, which were based on 1000 replications.

We developed a decision tree model to predict microbiologically confirmed tuberculosis, including variables from the best performing multivariable logistic regression model, using the classification and regression tree (CART) algorithm and *rpart* package (version 4.1-11) of the R statistical software. The CART algorithm builds a tree model through recursive partitioning, through which process the data is successfully split into increasingly homogenous subgroups. At each stage (also known as node), the algorithm selects a predictor and a cut-point associated with the best ability of the predictor to discriminate participants with tuberculosis from those without. This was less an issue in the current analyses with no continuous predictor. However, for class variables with more than two levels, the algorithm could collapse levels in order to achieve the best discrimination. The CART starts with one predictor, then adds other predictors (and nodes) until reaching homogenous groups or having subgroups with few participants (<5), or exhaustion of predictors which can contribute further to subgroups refinement. Due to the small size of the achieved tree, no pre- or post-pruning was applied. CART uses a generalization of the binomial variance (Gini index) for its impurity function, and employs a 10-fold cross-validation to estimate error rates. The algorithm code is available as *Extended data* ^30^.

## Results

### Study population

**Figure 1.**
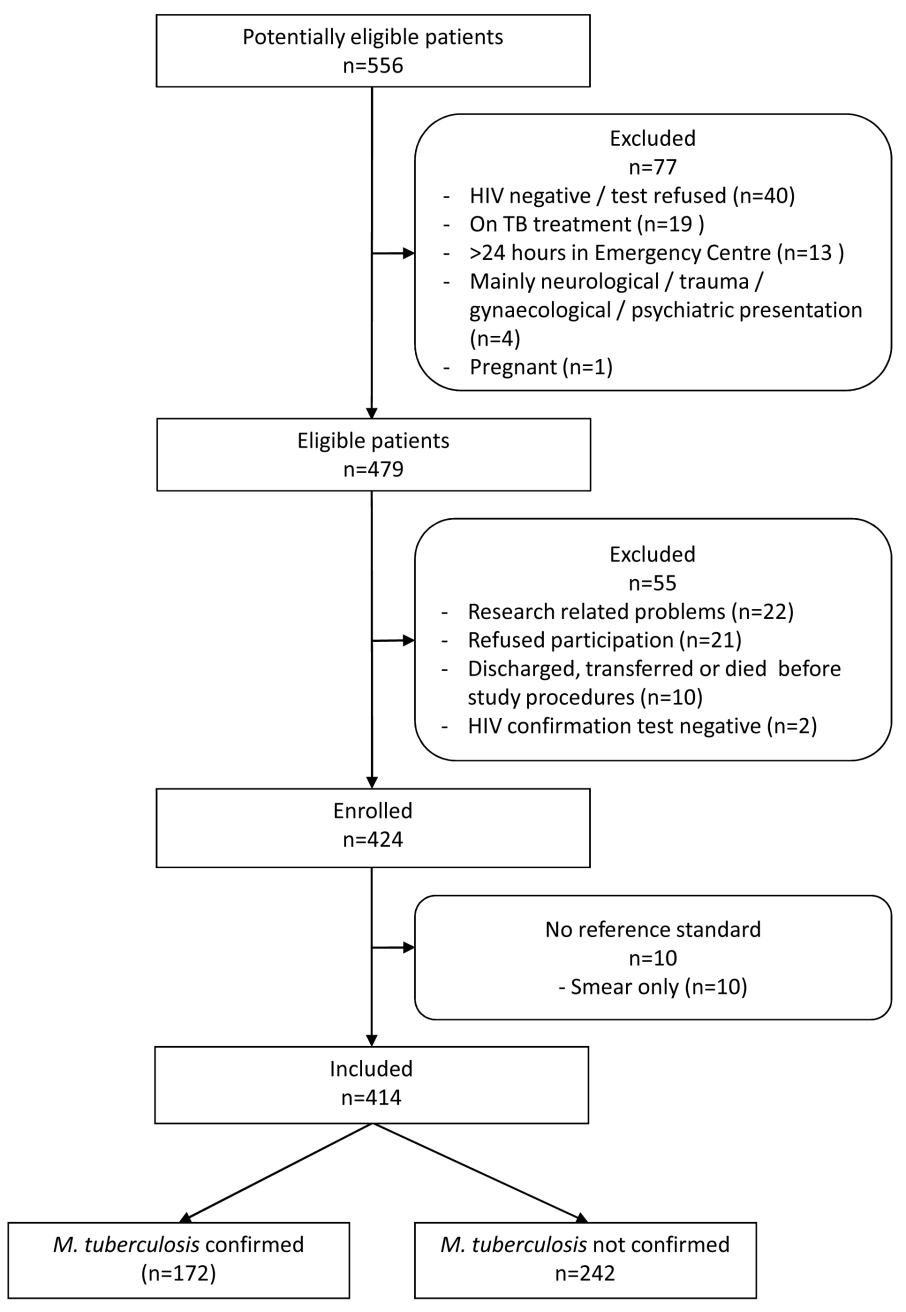
Flow diagram of study participants.

We screened 556 patients; 414 (74.5%) of whom were enrolled ( Figure 1). The prevalence of microbiologically confirmed tuberculosis was 41.5% (n=172): both Xpert MTB/RIF and culture positive n=93, 54.1%; only Xpert MTB/RIF positive n=32, 18.6%; only culture positive n=47, 27.3%. A median of 3 samples (25 ^th^–75 ^th^ percentile, 2–4) were obtained from participants for culture and/or Xpert MTB/RIF ( Table 1). At least two samples were obtained from two or more different anatomic sites in 350 (84.5%) participants.

**Table 1.** Total clinical samples sent for mycobacterial testing (includes ‘research’ and ‘routine’ samples).

Sample	Sample Samples obtained within 24 hours						Total samples obtained during admission					
	No. (%) patients producing ≥ 1 sample	Total no. samples	Total no. Xpert tests done	Total no. cultures done	No. positive culture and Xpert tests (%)	No. (%) TB patients with ≥1 positive culture or Xpert test	No. (%) patients producing ≥ 1 sample	Total no. samples	Total no. Xpert tests done	Total no. cultures done	No. positive culture and Xpert tests (%)	No. (%) TB patients with ≥1 positive culture or Xpert test
Urine	266 (69.5)	278	277	1	57 (20.5)	55 (37.2)	312 (75.4)	328	322	6	64 (19.5)	62 (36.0)
Sputum *	227 (59.3)	349	289	232	202 (38.8)	88 (59.5)	291 (70.3)	581	466	441	291 (32.2)	112 (65.1)
Blood	312 (81.5)	343	0	343	66 (19.2)	61 (41.2)	345 (83.3)	404	0	404	72 (17.8)	64 (37.2)
Fine needle aspirate (FNA)	4 (1.0)	4	2	4	5 (83.3)	3 (2.0)	10 (2.4)	12	5	6	9 (81.8)	5 (2.9)
Cerebrospinal fluid (CSF)	13 (3.4)	13	12	4	0 (0.0)	0 (0.0)	31 (7.5)	32	27	13	0 (0.0)	0 (0.0)
Pleural fluid	15 (3.9)	16	3	14	8 (47.1)	8 (5.4)	24 (5.8)	27	4	26	13 (44.8)	12 7.0)
Pericardial fluid	0 (0)	0	0	0	0 (0.0)	0 (0.0)	2 (0.5)	2	0	2	2 (100)	2 (1.2)
Ascitic fluid	1 (0.3)	1	0	1	1 (100)	1 (0.7)	2 (0.5)	2	0	2	1 (50.0)	1 (0.6)
Other (swab, tracheal aspirate)	1 (0.3)	1	1	1	0 (0.0)	0 (0.0)	2 (0.5)	2	1	2	0 (0.0)	0 (0.0)
	383 (92.5)	1005	584	600	339 (28.6)	148 (86.0)	414 (100)	1390	825	902	452 (26.2)	172 (100)

TB = Tuberculosis *Culture and Xpert MTB/RIF done on sputum taken on the same day were counted as two samples

Demographic and clinical characteristics of participants with and without confirmed tuberculosis are presented in Table 2. The median CD4 cell count was 86 cells/mm ^3^ (25 ^th^–75 ^th^ percentile, 30–218). The alternative diagnoses and the reasons for a clinical tuberculosis diagnosis in participants without microbiologically confirmed tuberculosis are presented in Table 3 and Table 4. The all-cause in-hospital mortality was 7.2% (n=30), 15 of whom had confirmed tuberculosis (representing 8.7% in hospital). These individual-level data are available at Zenodo ^31^.

**Table 2.** Demographic and clinical characteristics of study population.

Characteristics at enrolment (n (%) unless otherwise specified)	All (N=414)	*M. tuberculosis* confirmed (N = 172)	*M. tuberculosis* not confirmed (N = 242)	p-value ^⁋^
Age (years) (Median (Q _1_-Q _3_))	36 (30 – 43)	35 (30 – 42)	36 (31 – 44)	0.12
Gender: Male	171 (41.3)	71 (41.3)	100 (41.3)	0.99
Current cough of any duration	352 (85.0)	148 (86.0)	204 (84.3)	0.62
WHO symptom screen ≥ 1 present	414 (100)	172 (100)	242 (100)	---
WHO symptom screen ≥ 2 present	347 (83.8)	152 (88.4)	195 (80.6)	0.03
WHO symptom screen ≥ 3 present	239 (57.7)	104 (60.5)	135 (55.8)	0.34
Currently on antiretroviral therapy	195 (47.1)	62 (36.0)	133 (55.0)	<0.01
WHO danger signs ≥ 1 present	320 (77.3)	138 (80.2)	182 (75.2)	0.22
WHO danger signs ≥ 2 present	170 (41.1)	74 (43.0)	96 (39.7)	0.49
WHO danger signs ≥ 3 present	61 (14.7)	24 (14.0)	37 (15.3)	0.71
PoCUS individual features ≥ 1 present	264 (63.8)	133 (77.3)	131 (54.1)	<0.01
PoCUS individual features ≥ 2 present	140 (33.8)	86 (50.0)	54 (22.3)	<0.01
PoCUS individual features ≥ 3 present	64 (15.5)	47 (27.3)	17 (7.0)	<0.01
PoCUS independent features ≥ 1 present	217 (52.4)	116 (67.4)	101 (41.7)	<0.01
PoCUS independent features ≥ 2 present	80 (19.3)	58 (33.7)	22 (9.1)	<0.01
PoCUS independent features ≥ 3 present	17 (4.1)	17 (9.9)	0 (0.0)	<0.01
Urine lateral flow lipoarabinomannan (LF-LAM) positive *	94 (22.9)	71 (41.8)	23 (9.5)	<0.01
Hemoglobin (g/dl) (Mean ± SD) ^#^	9.7 ± 2.7	9.0 ± 2.4	10.2 ± 2.7	<0.01
CD4 cell count < 100 cells/mm ^3^^†^	219 (53.7)	108 (63.9)	111 (46.4)	<0.01
CD4 cell count 100 to 200 cells/mm ^3^^†^	77 (18.6)	32 (18.6)	45 (18.6)	1.0
CD4 cell count > 200 cells/mm ^3^^†^	117 (28.3)	32 (18.6)	85 (35.1)	<0.01
Chest x-ray: Possible tuberculosis	109 (26.3)	39 (22.7)	70 (28.9)	0.16
Chest x-ray: Likely tuberculosis	150 (36.2)	98 (57.0)	52 (21.5)	<0.01

SD = Standard Deviation; Q1-Q3 = 25th – 75th percentile; WHO symptom screen = Cough of any duration, fever, drenching night sweats, weight loss; WHO danger signs = Respiratory rate > 30/min, Heart rate > 120/min, Temperature > 39°C, being unable to walk unaided; PoCUS = Point-of-Care Ultrasound; PoCUS individual features = Pericardial effusion (any size), pleural effusion, ascites, any splenic lesion, intra-abdominal lymphadenopathy (any size); PoCUS independent features = Pericardial effusion (any size), ascites, intra-abdominal lymphadenopathy (any size); * N=411; ^#^ N=410; ^†^ N=408; ^⁋^ Comparison between *M. tuberculosis* confirmed and *M. tuberculosis* not confirmed

**Table 3.** Distribution of alternative diagnoses in participants without microbiologically confirmed tuberculosis.

Alternative diagnoses	n (%)
Lower Respiratory Tract Infection / Pneumonia	96 (39.7)
Clinical diagnoses of tuberculosis	63 (26.0)
Gastro-enteritis (acute & chronic)	13 (5.4)
Pneumocystis pneumonia	11 (4.5)
Renal failure (acute & chronic)	8 (3.3)
Bronchiectasis	6 (2.5)
Dysentery	3 (1.2)
HIV wasting syndrome	3 (1.2)
Undifferentiated abdominal pain	3 (1.2)
Appendicitis	2 (0.8)
Congestive cardiac failure	2 (0.8)
Delirium	2 (0.8)
Empyema	2 (0.8)
Gallstones	2 (0.8)
Kaposi sarcoma	2 (0.8)
Urosepsis	2 (0.8)
Bronchitis	1 (0.4)
Chronic Lymphocytic Leukaemia	1 (0.4)
Colon carcinoma	1 (0.4)
Constipation	1 (0.4)
Chronic Obstructive Pulmonary Disease (COPD) exacerbation	1 (0.4)
Cor Pulmonale	1 (0.4)
Duodenitis	1 (0.4)
*E. coli* bacteraemia	1 (0.4)
Interstitial lung disease	1 (0.4)
Liver carcinoma	1 (0.4)
Lung abscess	1 (0.4)
Meningitis	1 (0.4)
Non-tuberculous mycobacterial infection (disseminated)	1 (0.4)
Pelvic inflammatory disease	1 (0.4)
Progressive multifocal leukoencephalopathy	1 (0.4)
Scleroderma	1 (0.4)
Thrombotic thrombocytopenic purpura	1 (0.4)
Vitamin B12 deficiency	1 (0.4)
Unknown diagnosis	4 (1.7)
	242 (100)

HIV = Human Immunodeficiency Virus

**Table 4.** Reason for diagnosis of tuberculosis without microbiological confirmation.

Diagnostic test	n
Suggestive formal abdominal ultrasound done in radiology department	19
Suggestive chest X-ray	9
Positive urine lateral flow lipoarabinomannan (LF-LAM)	7
Suggestive formal abdominal ultrasound and suggestive chest X-ray	6
Not improving on empiric antibiotics	4
Raised adenosine deaminase (ADA) in effusion fluid (pleural or ascitic)	4
Cerebrospinal fluid suggestive of tuberculous meningitis (TBM)	4
Suggestive chest X-ray and positive urine LF-LAM	3
Suggestive formal abdominal ultrasound and positive urine LF-LAM	2
Psoas abscess on formal ultrasound	2
Caseous necrosis on biopsy (histology)	1
Suggestive computer tomography (CT) scan of abdomen	1
Suggestive chest X-ray and raised ADA in effusion fluid	1
Total	63

### Univariable associations

Univariable associations between microbiologically confirmed tuberculosis and clinical variables are presented as odds ratio (OR) with 95% CI and summarized in Table 5. The presence of two or more WHO screening symptoms (1.83 (1.04–3.22)), one or more PoCUS individual features (2.89 (1.87-4.47)), one or more PoCUS independent features (2.89 (1.92-4.35)), urinary LF-LAM (6.70 (3.99-11.25)), current antiretroviral therapy use (0.46 (0.31-0.68)), CD4 cell count less than 100 cells/mm ^3^ (1.98 (1.32-2.95)), and chest x-ray reported as ‘likely tuberculosis’ (4.81 (3.13-7.40)) were significantly associated with confirmed tuberculosis.

**Table 5.** Univariable associations between microbiologically confirmed tuberculosis and clinical variables.

Variables	Subgroups	Odds Ratio (95% CI)	p-value	AUC	AIC	-2 Log L	Likelihood ratio (χ ^2^ (p-value)	Calibration (χ ^2^ (p-value)
Intercept only			0.0006		564.03	562.03		
Presence of cough		1.15 (0.66-2.00)	0.623	0.509	565.79	561.79	0.243 (0.622)	NA
WHO symptoms screen	1 (reference)	1.00	0.128	0.540	562.65	556.65	4.312 (0.116)	0.0 (>0.999)
	2	1.84 (0.96-3.52)						
	3 or more	1.77 (0.99-3.18)						
	≥ 2 vs. < 2	1.83 (1.04-3.22)	0.036	0.539	561.39	557.39	4.645 (0.031)	NA
	≥ 3 vs. < 3	1.21 (0.81-1.80)	0.342	0.523	565.13	561.13	0.904 (0.342)	NA
WHO danger signs	Absent (reference)	1.00	0.511	0.542	566.63	558.63	2.327 (0.507)	0.0 (>0.999)
	1	1.31 (0.77-2.23)						
	2	1.52 (0.86-2.68)						
	3 or more	1.14 (0.59-2.22)						
	≥ 1 vs. < 1	1.34 (0.83-2.15)	0.230	0.525	564.57	560.57	1.462 (0.226)	NA
	≥ 2 vs. < 2	1.15 (0.77-1.71)	0.494	0.517	565.57	561.57	0.467 (0.474)	NA
	≥ 3 vs. < 3	0.90 (0.52-1.57)	0.706	0.507	565.89	561.89	0.143 (0.705)	NA
PoCUS individual features	Absent (reference)	1.00	<0.0001	0.679	522.43	514.43	46.52 (<0.0001)	0.0 (>0.999)
	1	1.74 (1.04-2.91)						
	2	3.08 (1.72-5.52)						
	3 or more	7.87 (4.05-15.28)						
	≥ 1 vs. < 1	2.89 (1.87-4.47)	<0.0001	0.616	541.88	537.88	24.149 (<0.0001)	NA
	≥ 2 vs. < 2	3.48 (2.27-5.33)	<0.0001	0.638	531.65	527.65	34.387 (<0.0001)	NA
	≥ 3 vs. < 3	4.98 (2.74-9.03)	<0.0001	0.602	534.32	530.32	31.71 (<0.0001)	NA
PoCUS independent features	Absent (reference)	1.00	<0.0001	0.673	510.29	502.29	58.66 (<0.0001)	0.0 (>0.999)
	1	1.87 (1.18-2.96)						
	2	4.69 (2.57-8.56)						
	3 or more	>999.99 (<0.001-999.99)						
	≥ 1 vs. < 1	2.89 (1.92-4.35)	<0.0001	0.629	538.98	534.98	27.05 (<0.0001)	NA
	≥ 2 vs. < 2	5.09 (2.96-8.73)	<0.0001	0.623	526.90	522.90	39.13 (<0.0001)	NA
	≥ 3 vs. < 3	>999.99 (<0.001-999.99)	0.975	0.549	535.14	531.14	30.89 (<0.0001)	NA
Urinary LAM		6.70 (3.99-11.25)	<0.0001	0.663	506.21	502.21	59.82 (<0.0001)	NA
Hemoglobin (per unit lower)		0.999 (0.996-1.001)	0.247	0.376	564.51	560.51	1.518 (0.218)	24.78 (0.0017)
Antiretroviral therapy status		0.46 (0.31-0.68)	0.0001	0.596	550.07	546.07	14.89 (<0.0001)	NA
CD4 cell count	>200 cells/mm ^3^ (reference)	1.00	0.0006	0.599	551.39	545.39	15.57 (0.0004)	0.0 (>0.999)
	<100 cells/mm ^3^	2.58 (1.59-4.20)						
	100-200 cells/mm ^3^	1.89 (1.03-3.47)						
Chest X-ray	Unlikely TB ^g^ (reference)	1.00	<0.0001	0.704	506.473	500.47	60.48 (<0.0001)	0.0 (>0.999)
	Possible TB	1.94 (1.12-3.34)						
	Likely TB	6.46 (3.90-10.70)						
	Possible TB vs Unlikely & Likely TB	0.73 (0.46-1.15)	0.175	0.530	563.09	559.09	1.86 (0.172)	
	Likely TB vs. Unlikely and Likely TB	4.81 (3.13-7.40)	<0.0001	0.677	510.18	506.18	54.78 (<0.0001)	NA
	Possible & Likely TB vs. Unlikely TB	3.86 (2.44-6.12)	<0.0001	0.641	528.04	524.04	33.16 (<0.0001)	NA

CI = Confidence Interval; AUC = Area under the receiver operating characteristics curves; AIC = Akaike information criterion; WHO symptom screen = Cough of any duration, fever, drenching night sweats, weight loss; WHO danger signs = Respiratory rate > 30/min, Heart rate > 120/min, Temperature > 39°C, being unable to walk unaided; PoCUS = Point-of-Care Ultrasound; PoCUS individual features = Pericardial effusion (any size), pleural effusion, ascites, any splenic lesion, intra-abdominal lymphadenopathy (any size); PoCUS independent features = Pericardial effusion (any size), ascites, intra-abdominal lymphadenopathy (any size); LAM = Lateral flow lipoarabinomannan; TB = tuberculosis

### Multivariable model

Measures of model performance are summarized in Table 6. The initial model (WHO screening symptoms ≥2, antiretroviral therapy use) had poor discriminatory power in predicting confirmed tuberculosis with an AUC of 0.615. The addition of either PoCUS independent features or PoCUS individual features to the initial model both improved model goodness of fit and its discriminatory power, however the model with PoCUS independent features had a greater AUC and a smaller AIC. The further addition of urinary LF-LAM and chest x-ray improved the model. Adding CD4 cell count did not improve the performance of the model ( Table 6).

**Table 6.** The performance of multivariable models predicting microbiologically confirmed tuberculosis.

Model	Variables in the model	AUC (95% CI)	AIC	-2 Log L	Likelihood ratio χ ^2^	Calibration χ ^2^ (p-value)
A	WHO symptom screen ≥2, ART use	0.615 (0.564-0.665)	547.80	541.80	18.12 (<0.0001) DF2	1.386 (0.500)
B	A + PoCUS independent features	0.703 (0.653-0.753)	503.66	491.66	69.30 (<0.0001) DF5	4.330 (0.741)
C	A + PoCUS individual features	0.701 (0.650-0.751)	514.26	502.26	58.70 (<0.0001) DF5	5.391 (0.612)
D	A + urinary LAM	0.736 (0.688-0.784)	489.64	481.64	79.32 (<0.0001) DF3	3.981 (0.408)
E	D + PoCUS independent features	0.773 (0.727-0.819)	463.38	449.38	111.57 (<0.0001) DF6	5.259 (0.628)
F	E + Chest x-ray	0.820 (0.779-0.862)	433.99	415.99	144.966 (<0.0001) DF8	7.429 (0.386)
G	F + CD4 cell count	0.821 (0.780-0.863)	435.99	413.99	146.970 (<0.0001) DF10	8.753 (0.363)

p-values for AUC comparisons: 0.0009 (B-A), 0.0001 (C-A), <0.0001 (D-A), <0.0001 (E-A), <0.0001 (F-A), <0.0001 (G-A); 0.868 (C-B), 0.237 (D-B), 0.0003 (E-B), <0.0001 (F-B), <0.0001 (G-B), 0.181 (D-C), 0.0002 (E-C), <0.0001 (F-C), <0.0001 (G-C), 0.0052 (E-D), <0.0001 (F-D), <0.0001 (G-D), 0.002 (F-E), 0.0022 (G-E), 0.8223 (G-F); Overall p<0.0001 for difference across all AUC.

AUC = Area under the receiver operating characteristics curves; CI = Confidence interval; AIC = Akaike information criterion; WHO symptom screen = Cough of any duration, fever, drenching night sweats, weight loss; ART = Antiretroviral therapy; PoCUS = Point-of-Care Ultrasound; PoCUS individual features = Pericardial effusion (any size), pleural effusion, ascites, any splenic lesion, intra-abdominal lymphadenopathy (any size); PoCUS independent features = Pericardial effusion (any size), ascites, intra-abdominal lymphadenopathy (any size); LAM = Lateral flow lipoarabinomannan

Based on RIDI% estimates, adding urinary LF-LAM, PoCUS independent features, and chest x-ray to the initial and subsequent models conferred similar levels of improvement for tuberculosis prediction ( Table 7). Change in RIDI% was meaningless when CD4 cell count was added to the model comprising WHO symptoms screen, antiretroviral therapy use, PoCUS independent features, urinary LF-LAM and chest X-ray (RIDI% 2.6 (2.4-2.7)).

**Table 7.** Relative integrated discrimination improvement (RIDI, %) statistic comparing different models.

Model	Variables in the model	A	B	C	D	E	F	G
A	WHO symptom screen ≥2, ART use	NA	252.6 (242.3-262.8)	224.4 (214.8-233.9)	334.5 (321.0-348.0)	483.0 (464.6-501.5)	653.9 (629.5-678.3)	674.2 (649.0-699.4)
B	A + PoCUS independent features		NA	-7.77 (-8.37 to -7.17)	25.3 (23.5-27.1)	65.0 (63.4-66.5)	113.4 (111.1-115.8)	119.0 (116.6-121.4)
C	A + PoCUS individual features			NA	37.1 (35.0-39.3)	80.7 (78.6-82.8)	133.7 (130.8-136.6)	139.8 (136.8-142.8)
D	A + urinary LAM				NA	34.3 (33.4-35.1)	73.8 (72.3-75.3)	78.4 (76.8-80.0)
E	D + PoCUS independent features					NA	29.4 (28.7-30.1)	32.8 (32.0-33.5)
F	E + Chest x-ray						NA	2.6 (2.4-2.7)
G	F + CD4 cell count							NA

WHO symptom screen = Cough of any duration, fever, drenching night sweats, weight loss; ART = Antiretroviral therapy; PoCUS = Point-of-Care Ultrasound; PoCUS individual features = Pericardial effusion (any size), pleural effusion, ascites, any splenic lesion, intra-abdominal lymphadenopathy (any size); PoCUS independent features = Pericardial effusion (any size), ascites, intra-abdominal lymphadenopathy (any size); LAM = Lateral flow lipoarabinomannan.

### Prediction tree

**Figure 2.**
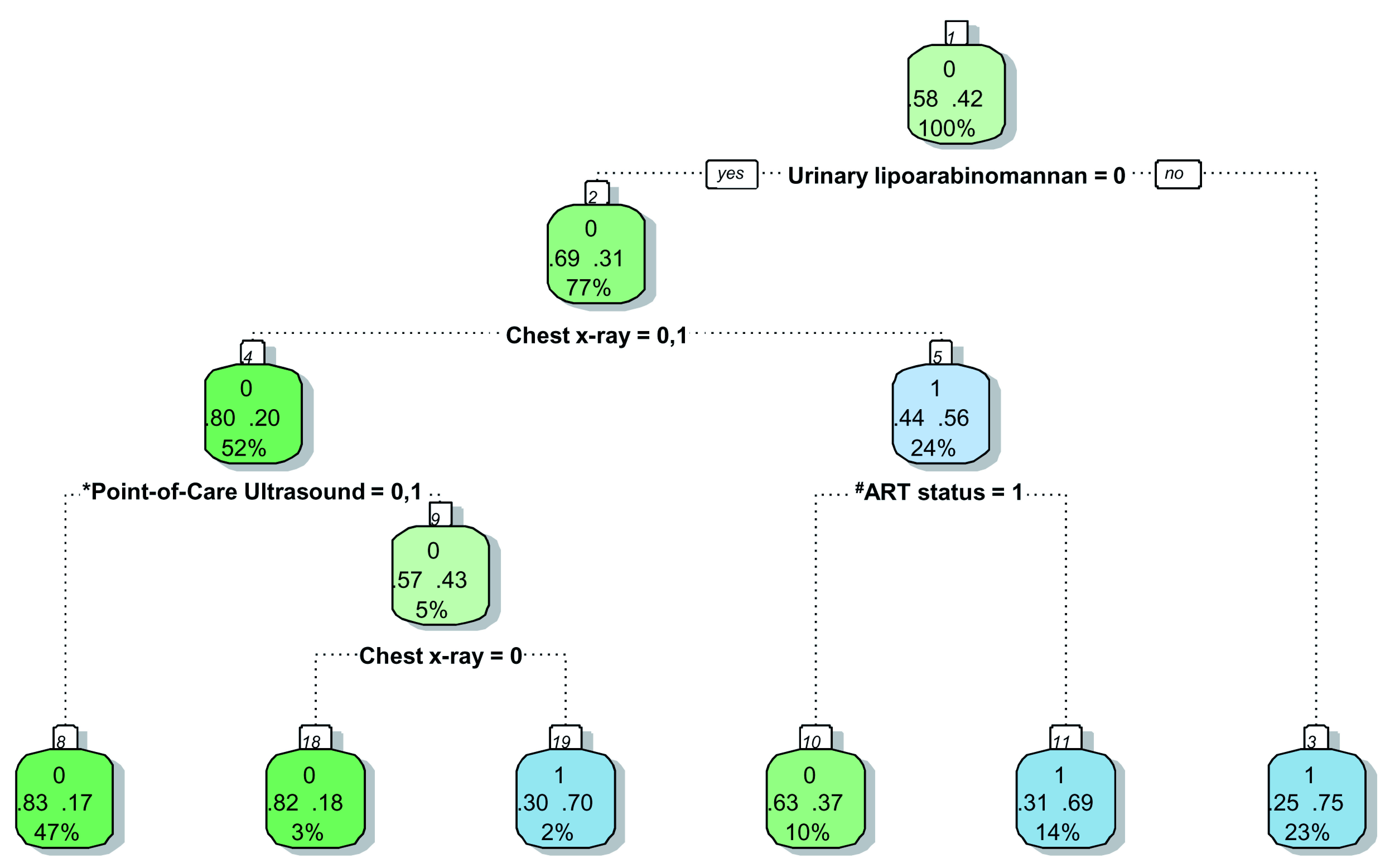
Prediction tree for microbiologically confirmed tuberculosis. Totals might not add up due to rounding. *Point-of-Care ultrasound = Independent point-of-care ultrasound features (ascites, any size pericardial effusion, any size intra-abdominal lymphadenopathy; ^#^ART = Anti-retroviral therapy. Predictor coding: Urinary lipoarabinomannan: 0 = Negative, 1 = Positive; Chest x-ray: 0 = Unlikely tuberculosis, 1 = Possible tuberculosis, 2 = Likely tuberculosis; Point-of-Care ultrasound: 0 = None present; 1 = ≥1 feature present, 2 = ≥2 features present; ART status: 0 = Not on ART, 1 = Currently on ART. Explanation of node: Number in small white block represents the number of the node in the recursive partitioning; Bottom number in big coloured block represents the percentage of the entire dataset that passes through this particular node (e.g. 100% in block 1); Middle numbers in big coloured block represent the proportion with the outcome (right) and without the outcome (left) within the subgroup (e.g. in block, 58% without tuberculosis and 42% with tuberculosis in block1); Top number in big coloured block represent the presence (1) or absence (0) of the outcome in the majority of the observations in that block (e.g. majority in block 1 without tuberculosis (58% versus 42%)); The colour of the block has no particular meaning.

**Figure 3.**
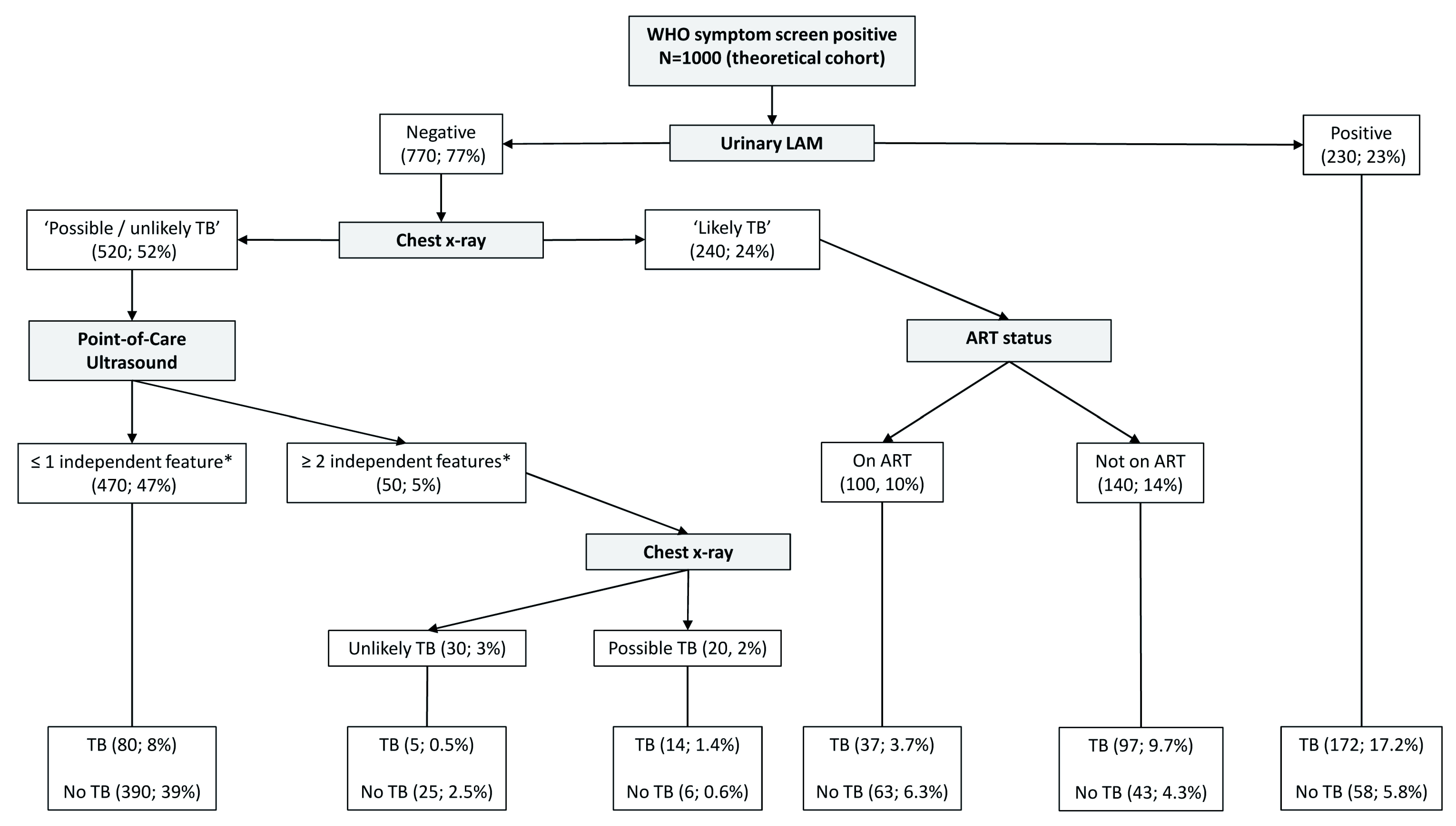
Prediction tree for microbiologically confirmed tuberculosis applied to a theoretical cohort of 1000 patients. Nodes show the number of patients and percentage of total sample size (n, %). Totals might not add up due to rounding. *Independent point-of-care ultrasound features (ascites, any size pericardial effusion, any size intra-abdominal lymphadenopathy; LAM = lateral flow lipoarabinomannan; TB = tuberculosis; ART = anti-retroviral therapy.

**Figure 4.**
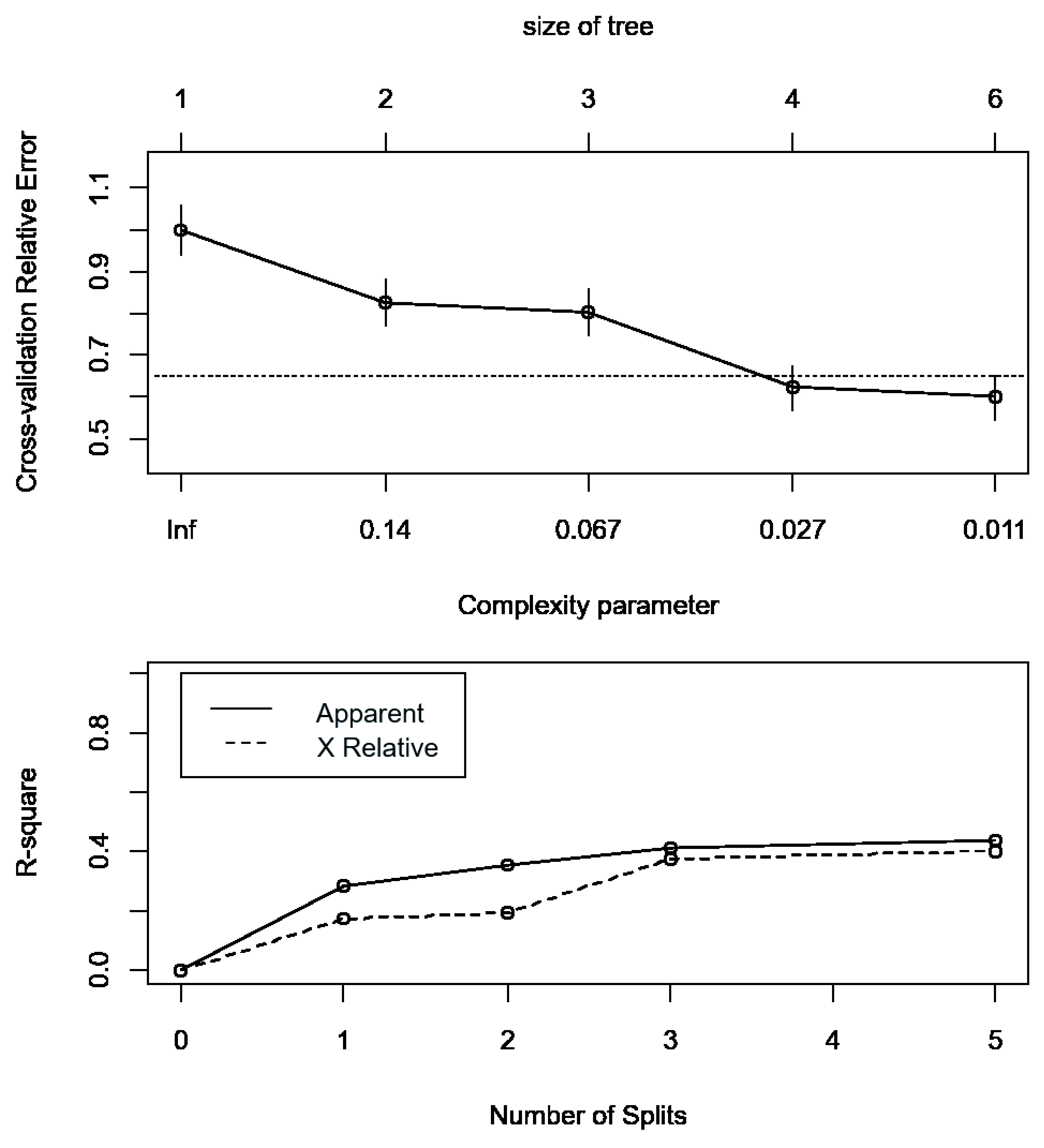
Cross-validation results of the decision tree. Upper panel: cross-validation relative error vs. numbers of split and complexity parameter; Lower panel: apparent R-square and R-square from cross-validation vs the number of splits.

Significant variables (Model F in Table 7) were included in the splitting process to build the classification tree for microbiologically confirmed tuberculosis. The CART created for confirmed tuberculosis is shown in Figure 2, and the CART as applied to a theoretical cohort of 1000 patients is presented in Figure 3. The CART analysis suggest that once screened via WHO symptoms as eligible for further diagnostic investigations, the number of WHO symptoms present does not add further to the discrimination of people with tuberculosis from those without. Furthermore, CART positions urinary LF-LAM as the next screening test after WHO symptoms, with 75% of people with positive urinary LF-LAM test (17% of all those with positive WHO symptoms) having a definitive diagnosis of microbiologically confirmed tuberculosis ( Figure 2 and Figure 3). For those with negative urinary LF-LAM, CART positions chest x-ray as the next screening test. Chest x-ray appears twice, but with complementary and not overlapping contributions. The first appearance of chest x-ray (after those with negative urinary LF-LAM) serves to separate participants with ‘likely tuberculosis’ on chest x-ray from those with ‘possible or unlikely tuberculosis’ on chest x-ray. The presence of one or no PoCUS independent features in those with ‘possible or unlikely tuberculosis’ on chest x-ray (47% of the starting sample) isolates 83% of this subgroup (representing 39% of the starting sample) where tuberculosis was not microbiologically confirmed ( Figure 2 and Figure 3). The second appearance of chest x-ray occurs in participants with ≥2 PoCUS independent features and serves to separate those with ‘possible tuberculosis’ on chest x-ray from those with ‘unlikely tuberculosis’ on chest x-ray. The validation for the decision tree is presented in Figure 4.

**Figure 5.**
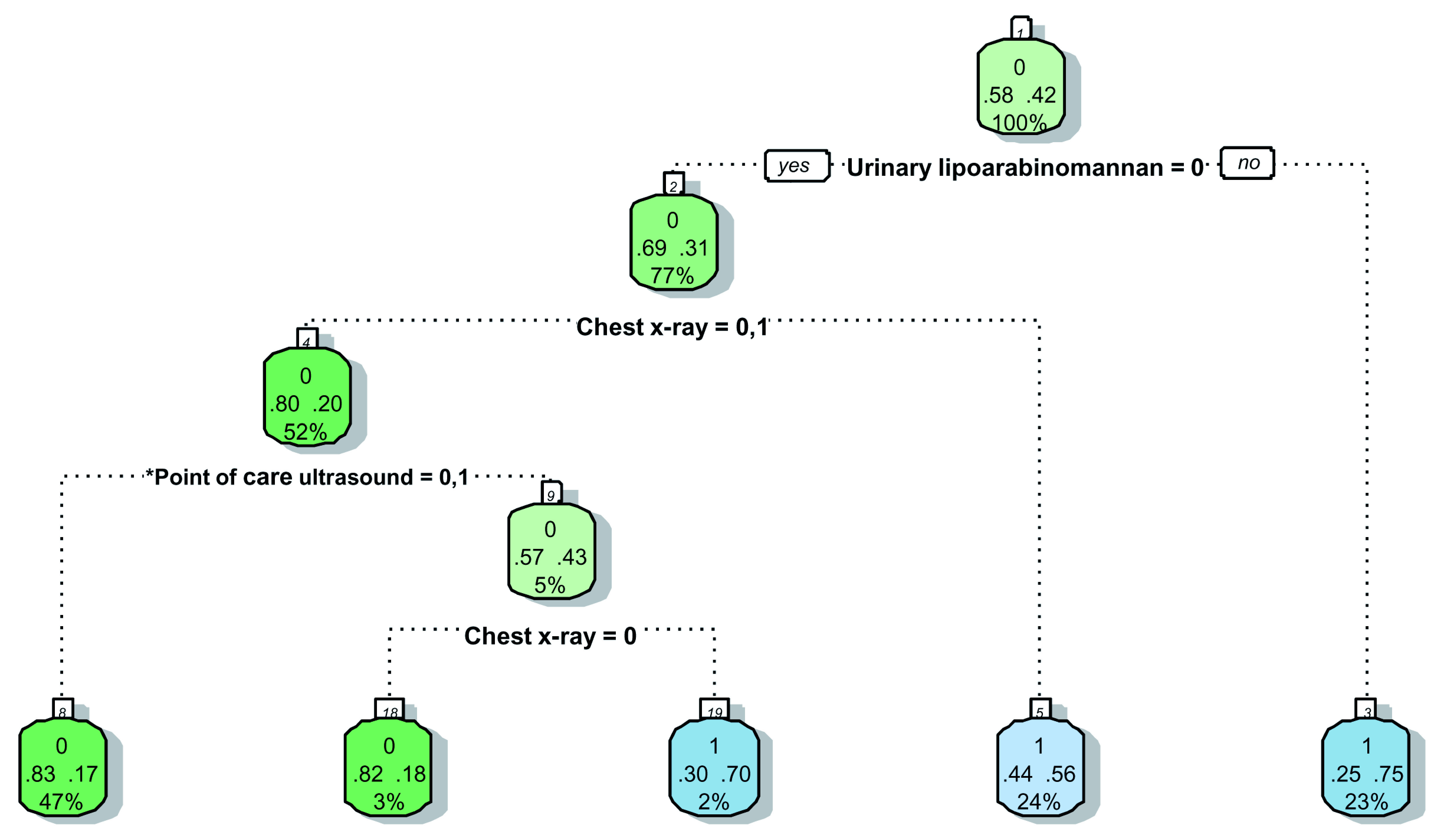
Prediction tree with antiretroviral status removed for microbiologically confirmed tuberculosis. Totals might not add up due to rounding. *Point-of-Care ultrasound = Independent point-of-care ultrasound features (ascites, any size pericardial effusion, any size intra-abdominal lymphadenopathy. Predictor coding: Urinary lipoarabinomannan: 0 = Negative, 1 = Positive; Chest x-ray: 0 = Unlikely tuberculosis, 1 = Possible tuberculosis, 2 = Likely tuberculosis; Point-of-Care ultrasound: 0 = None present; 1 = ≥1 feature present, 2 = ≥2 features present. Explanation of node: Number in small white block represents the number of the node in the recursive partitioning; Bottom number in big coloured block represents the percentage of the entire dataset that passes through this particular node(e.g. 100% in block 1); Middle numbers in big coloured block represent the proportion with the outcome (right) and without the outcome (left) within the subgroup (e.g. in block, 58% without tuberculosis and 42% with tuberculosis in block1); Top number in big coloured block represent the presence (1) or absence (0) of the outcome in the majority of the observations in that block (e.g. majority in block 1 without tuberculosis (58% versus 42%)); The colour of the block has no particular meaning.

**Figure 6.**
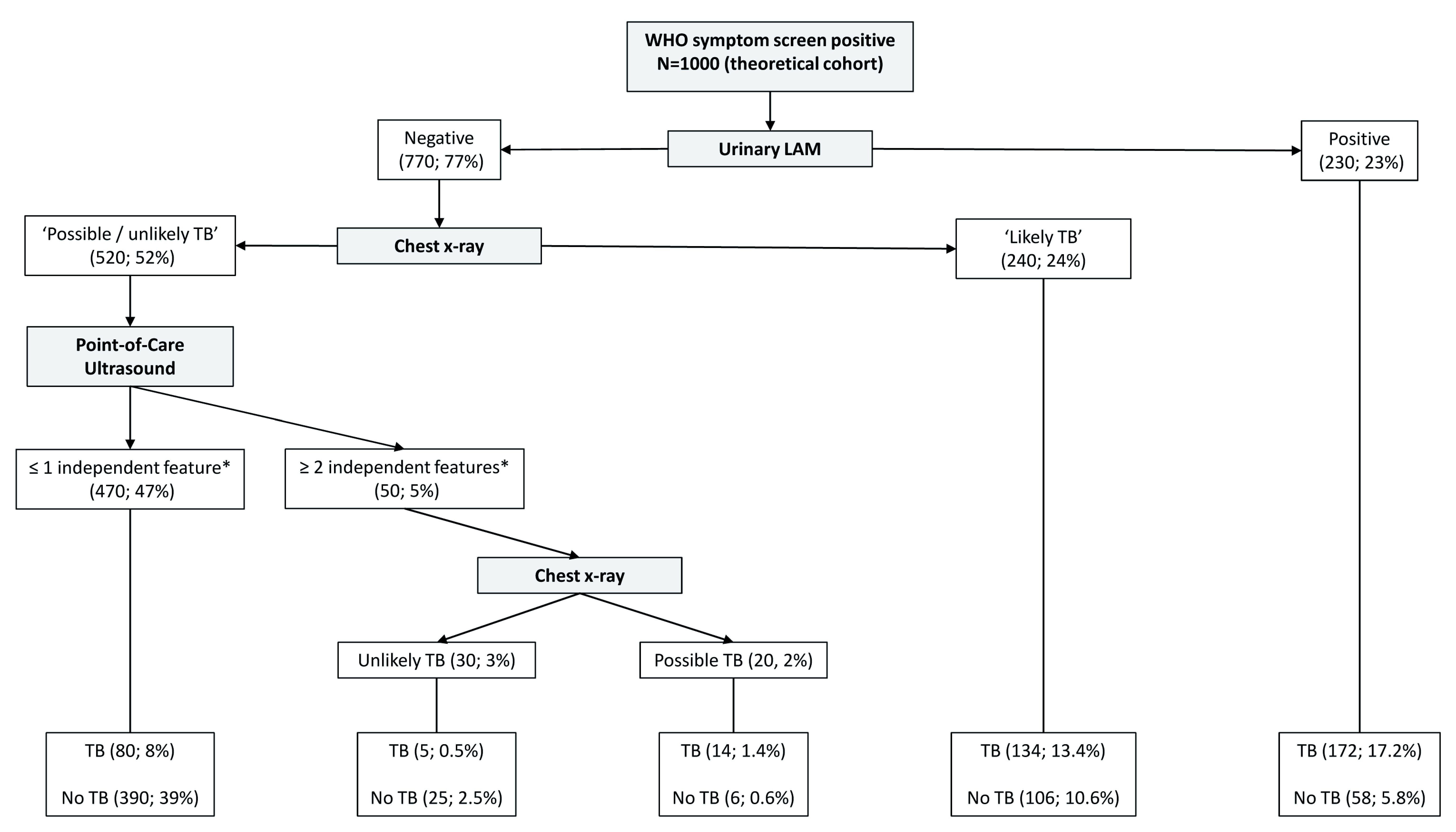
Prediction tree with antiretroviral status removed for microbiologically confirmed tuberculosis applied to a theoretical cohort. Nodes show the number of patients and percentage of total sample size (n, %). Totals might not add up due to rounding. *Independent point-of-care ultrasound features (ascites, any size pericardial effusion, any size intra-abdominal lymphadenopathy; LAM = Lateral flow lipoarabinomannan; TB = tuberculosis.

We created a second decision tree to make it more clinically applicable by removing the history of antiretroviral therapy (ART) status, because ART interruption is often not disclosed and ART status may be unavailable in confused patients ( Figure 5 and Figure 6). The branch on the original tree relating to antiretroviral therapy no longer expands, narrowing down what to decide for the 24% of the sample with negative urinary LF-LAM and ‘likely tuberculosis’ on chest x-ray. Just over half (56%) of these participants will have confirmed tuberculosis.

**Figure 7.**
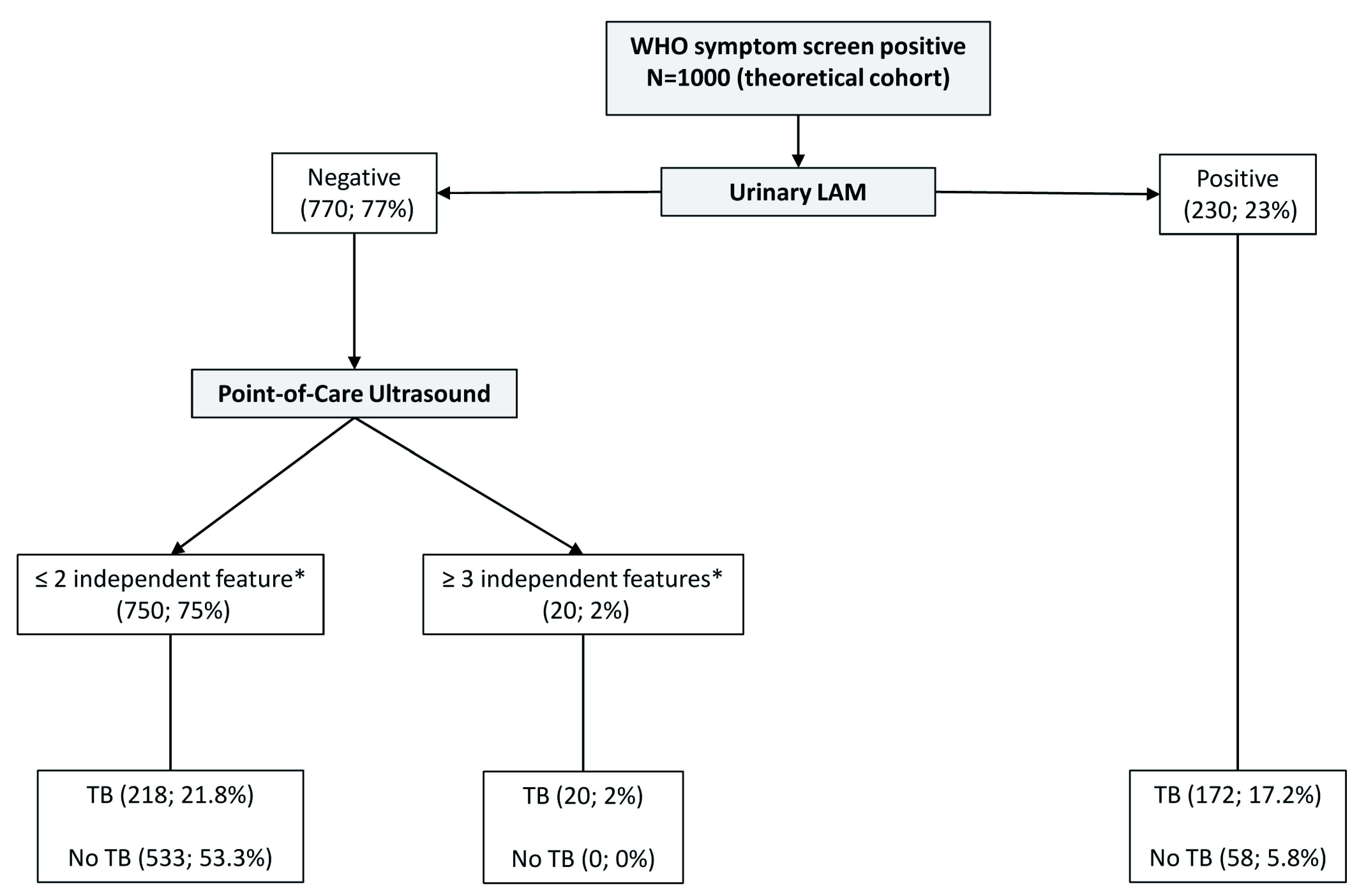
Prediction tree of point-of-care only tests for microbiologically confirmed tuberculosis applied to a theoretical cohort. Nodes show the number of patients and percentage of total sample size (n, %). Totals might not add up due to rounding. *Independent point-of-care ultrasound features (ascites, any size pericardial effusion, any size intra-abdominal lymphadenopathy); LAM = lateral flow lipoarabinomannan; TB = tuberculosis.

**Figure 8.**
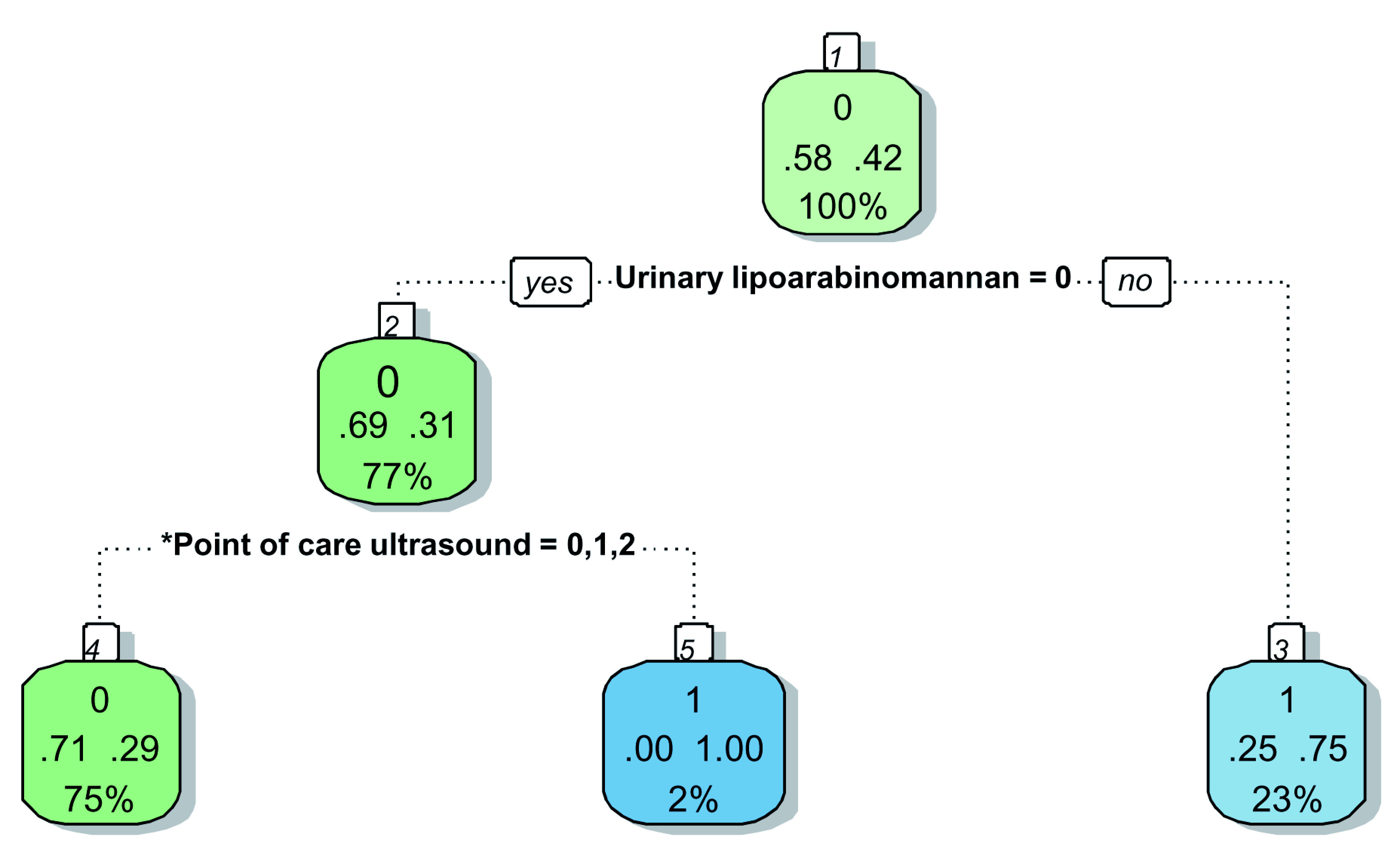
Prediction tree with chest x-ray removed for microbiologically confirmed tuberculosis. *Point-of-Care ultrasound = Independent point-of-care ultrasound features (ascites, any size pericardial effusion, any size intra-abdominal lymphadenopathy). Predictor coding: Urinary lipoarabinomannan: 0 = Negative, 1 = Positive; Point-of-Care ultrasound: 0 = Negative; 1 = ≥1 feature present, 2 = ≥2 features present, 3 = ≥3 features present. Explanation of node: Number in small white block represents the number of the node in the recursive partitioning; Bottom number in big coloured block represents the percentage of the entire dataset that passes through this particular node(e.g. 100% in block 1); Middle numbers in big coloured block represent the proportion with the outcome (right) and without the outcome (left) within the subgroup (e.g. in block, 58% without tuberculosis and 42% with tuberculosis in block1); Top number in big coloured block represent the presence (1) or absence (0) of the outcome in the majority of the observations in that block (e.g. majority in block 1 without tuberculosis (58% versus 42%)); The colour of the block has no particular meaning.

We created a third prediction tree by only excluding chest x-ray, which is not a true point-of-care test ( Figure 7 and Figure 8). CART positions PoCUS as the next screening test for those with a negative urinary LF-LAM. The presence of two or less independent PoCUS features (75% of the starting sample) had a true negative rate of 71% (representing 53% of the starting sample) in the subgroup where tuberculosis was not microbiologically confirmed.

## Discussion

We developed a prediction tree to diagnose HIV-associated tuberculosis in an emergency centre in a high burden setting. The variables selected on multivariable analysis for inclusion in the final model were the presence of >2 WHO screening symptoms, current antiretroviral therapy use, urinary LF-LAM, independent PoCUS features, and chest x-ray. The CART analysis positioned urinary LF-LAM as the first test to perform in participants with positive WHO screening symptoms, followed by chest x-ray. We also developed a simplified prediction tree by excluding chest x-ray, which is not a true point-of-care test: CART positioned PoCUS as the next screening test for those with a negative urinary LF-LAM.

The use of urinary LF-LAM was the predictor with the best ability of creating pure groups (either with or without tuberculosis); classifying almost 25% of the study sample (75% of which were true positives) regardless of their CD4 cell count. The false positive rate of 25% is less than a recent Cochrane review, in which 33% of participants with tuberculosis symptoms had a false positive urinary LF-LAM result for microbiologically confirmed tuberculosis ^32^. However, inappropriate exclusions (e.g. participants unable to produce sputum), different enrolment criteria and different CD4 cell counts could potentially explain the high false negative rate seen in the Cochrane review ^32^. Another urine-based LAM assay, Fujifilm SILVAMP TB LAM (FujiLAM; Fujifilm, Tokyo, Japan), has higher sensitivity but somewhat lower specificity than the LF-LAM assay we used ^33^.

Urinary LAM is underutilized despite it being affordable, fast, non-invasive, and simple ^34^. Only three high TB/HIV burden countries (Eswatini (formerly Swaziland), South Africa, and Uganda) had national roll-outs of LF-LAM testing by the end of 2018 ^34^. Urine LF-LAM is a simple point-of-care test achievable in acute care settings ^21^, which has been shown to reduce mortality in high-risk HIV-positive inpatients ^35, 36^.

The performance of PoCUS when chest x-ray is available is limited ( Figure 2 and Figure 3). One of every 11 PoCUS examinations will be ‘positive’ (i.e. two or more PoCUS independent features), but then an evaluation of the chest x-ray would still be needed to refine the classification of patients with and without tuberculosis. A ‘negative’ PoCUS examination (i.e. the presence of ≤1 PoCUS independent feature) will only rule out 39% of all patients with a clinical suspicion of tuberculosis. This supports other studies and the current WHO guidelines that ultrasound is an additional diagnostic tool and should not replace chest x-ray as the initial imaging step to diagnose tuberculosis in HIV-positive patients ^20, 37^. However, chest x-ray is not a true point-of-care test, unlike PoCUS. In acute care settings where chest x-ray is not readily available PoCUS has a 100% true positive rate when all 3 of the independent features were detected, indicating its potential value as a rule-in test; however, 39 PoCUS examinations will need to be performed to confidently diagnose one additional patient in those who had a negative LAM. The presence of ≤2 PoCUS independent features will rule out 53% of patients with a clinical suspicion of tuberculosis in situations where chest x-ray is not available; however, the high false negative rate (29%, 218/750) indicates that PoCUS cannot be used as a rule-out test and these patients will need to undergo further testing.

The use of urinary LF-LAM should be prioritised in all HIV-positive patients (regardless of CD4 cell count and clinical condition) who presents to the emergency centre with WHO tuberculosis symptoms. Although a result can be obtained after 25 minutes, a major time increasing factor would be to get a urine sample. The history of current use of ART should be obtained if the patient’s condition allows, as it further refines the diagnostic ability of the algorithm by increasing both the true positive and the true negative rate. Chest x-ray should still be performed if available. In these settings, the value of PoCUS becomes doubtful due to the low positive yield (5%) and the further interpretation of a chest x-ray to better classify cases and non-cases. Although 47% of patients will have negative results for urinary LF-LAM, chest x-ray and PoCUS, the true negative rate is only 83%, too low to confidently rule tuberculosis out. In emergency centres without chest x-ray availability (e.g. limited resources, restricted radiology consulting times), physicians can confidently diagnose tuberculosis in patients where all three independent PoCUS features are present (true positive rate 100%). However, only 2% of the PoCUS examinations are expected to be positive and one can argue whether the time spend to perform the PoCUS is worthwhile. The 71% true negative rate again indicates the need for further diagnostic testing.

Our study has some limitations. Our findings may not be generalizable as the study was conducted in a single emergency centre in a high TB/HIV-prevalence setting; a single, experienced operator performed all the PoCUS examinations; and the chest x-rays were interpreted by a single experienced radiologist. The main strength of our study is the robust microbiologic reference standard composed of TB culture and Xpert MTB/RIF performed on multiple samples from different anatomic sites. However, it is still possible that some TB cases were missed by the reference standard. The study was also performed under routine conditions experienced in the emergency centre. Lastly, robust analytic strategies were used to develop and validate the diagnostic decision tree.

## Conclusion

We developed a near-patient and point-of-care decision tree for the diagnosis of HIV-associated tuberculosis in acute care settings. Implementing this decision tree following screening via WHO symptoms can allow immediate initiation of TB treatment within the emergency centre in about a quarter of suspected patients among whom 75% would have microbiologically confirmed tuberculosis, or withhold such treatment in nearly half of suspected patients, among whom less than 18% will have microbiologically confirmed tuberculosis. Urinary LF-LAM had a 75% true positive rate, representing 17% of participants with positive WHO screening symptoms regardless of CD4 cell count and its use should be prioritised. The contribution of PoCUS in the context of urinary LF-LAM and chest X-ray availability was limited, due to the low positive yield, the need for further chest x-ray interpretation and the high false negative rate. In acute care settings without chest x-ray availability, PoCUS has a 100% true positive rate, but will only affect 2% of eligible patients. The role of PoCUS in diagnosing HIV-associated tuberculosis in the emergency centre needs to be further investigated.

## Data availability

### Underlying data

Zenodo: Rapid diagnosis of HIV-associated tuberculosis in the emergency centre. https://doi.org/10.5281/zenodo.3734101 ^31^.

This project contains the following underlying data:

- HIV-TB_diagnostic_algorithm_data.csv. (Data used for diagnostic algorithm.)
- HIV-TB_diagnostic_samples.csv. (Data of diagnostic samples taken.)
- HIV-TB_diagnostic_algorithm_codebook.docx. (codebook for diagnostic algorithm data.)
- HIV-TB_diagnostic_samples_codebook.docx. (codebook for diagnostic samples.)

### Extended data

Zenodo: Case report form: Rapid diagnosis of HIV-associated tuberculosis in the emergency centre. https://doi.org/10.5281/zenodo.3738912 ^23^.

Zenodo: Code: Rapid diagnosis of HIV-associated tuberculosis in the emergency centre. https://doi.org/10.5281/zenodo.3739005 ^30^.

### Reporting guidelines

Zenodo: TRIPOD checklist for ‘A multi-parameter diagnostic clinical decision tree for the rapid diagnosis of tuberculosis in HIV-positive patients presenting to an emergency centre’. https://doi.org/10.5281/zenodo.3738999 ^38^.

Data are available under the terms of the Creative Commons Zero "No rights reserved" data waiver (CCO 1.0 Public domain dedication).
