# Supplementary material for: A multi-parameter diagnostic clinical decision tree for the rapid diagnosis of tuberculosis in HIV-positive patients presenting to an emergency centre
Source: Wellcome Open Res. 2022 May 12;5:72. Originally published 2020 Apr 17. [Version 2] doi: 10.12688/wellcomeopenres.15824.2 (PMC9608260; doi:10.12688/wellcomeopenres.15824.2)
Supplement: Peer review history

# Peer review: Reviewer response for version 2

**DOI:** 10.21956/wellcomeopenres.19817.r51280

**Published:** 2022-06-30

**Authors:** Peter MacPherson

**Affiliations:** 1 Liverpool School of Tropical Medicine, Liverpool, UK

Thanks for asking me to review this manuscript. Very nice study, and clearly reported. I have only a few comments the authors should address.

 Major comments

1. Prevalence of microbiologically-confirmed TB is very high in this population, and health facilities seem to be reasonably well resourced. The authors should add text to the Discussion to discuss generalisbility to settings in Africa outside of the Khayelitsha area.
2. The CD4 cell count profile is low, and antiretroviral therapy coverage considerably lower than we have seen in other settings. It would be good to add a sentence in the discussion to reflect on how the accuracy, performance, and applicability of the final decision tree may be affected in other settings in the African continent where we see different patterns of ART coverage.
3. Presumably HIV viral load measurements were not available for inclusion in the model? We have seen in several studies now a moderately high prevalence of detectable viraemia in HIV-positive adults who reported taking ART, and is strongly associated with adverse clinical outcomes. I could imagine that HIV viral load measurement (e.g. using the GeneXpert platform) might be a useful diagnostic predictor of prevalent TB. It would be worth adding a sentence to discuss ART coverage and viral failure in the limitations section of the Discussion.
4. In Figure 1, not clear what were the reasons for “Research related problems” in the 55 participants who were excluded. The authors should add a sentence to describe these participants in more details in the results, and provide reassurance that these exclusions have not resulted in selection/spectrum bias.
5. Discussion. Sentence about coverage of urine LAM is a little outdated, and several more countries have rolled-out this test, including Malawi.
6. POCUS is critically dependent on trained operators and high levels of quality assurance, which are not usually available in most settings in Africa. In most settings, operators trained in POCUS will not be available, whereas digital chest with CAD is often available, meaning that pragmatically, the decision tree placement of POCUS prior to CXR doesn’t necessarily make sense. I would perhaps temper the paragraph about the utility of POCUS, especially given the suboptimal performance found here, and in other studies.

Minor Comments:

1. Abstract, typo: “Logistic regressions” should be “Logistic regression”.

Is the work clearly and accurately presented and does it cite the current literature?

Yes

If applicable, is the statistical analysis and its interpretation appropriate?

Yes

Are all the source data underlying the results available to ensure full reproducibility?

Yes

Is the study design appropriate and is the work technically sound?

Yes

Are the conclusions drawn adequately supported by the results?

Yes

Are sufficient details of methods and analysis provided to allow replication by others?

Yes

Reviewer Expertise:

HIV, TB, Public Health, epidemiology, randomised controlled trials, diagnostic accuracy evaluations, global health

I confirm that I have read this submission and believe that I have an appropriate level of expertise to confirm that it is of an acceptable scientific standard.

# Peer review: Reviewer response for version 1

**DOI:** 10.21956/wellcomeopenres.17356.r38643

**Published:** 2020-06-08

**Authors:** Maria Teresa Giordani

**Affiliations:** 1 Infectious and Tropical Diseases Department, San Bortolo Hospital, Vicenza, Italy

This manuscript is aimed to describe the use of LF-LAM in an acute care setting and identified PoCUS features independently associated with HIV-associated tuberculosis. Then, the study proposes a decisional algorithm for the diagnosis of TB in HIV patients, including point-of-care tests, as a result of a regression tree algorithm (CART).

 This algorithm combines the only two tests really available at the patient bedside and having the true characteristics of point of care test: LF-LAM and PoCUS, and the “classic” tests, CXR and GeneXpert PCR plus the culture of the sputum or pus: the two latter tests are now considered as the gold standard.

 The scenario is peculiar: the patients accessing this hospital have a high prevalence of HIV and are very deeply immunosuppressed, with very low CD4 count (never treated before for both conditions, or only treated for HIV).

**General comments**: Portable CXR and GeneXpert, reported in the text as “near point-of care-tests” really improved the opportunity of TB diagnosis in the last decades and now are the standard-of-diagnostic tools in many hospitals in many different resource settings. However, the clinical impact of these technologies on the TB epidemic and mortality was not so impressive, particularly if we consider patients living with HIV.

 The point-of-care tests like LF-LAM and PoCUS are available also in resource-limited settings (RLS) with affordable costs, can be performed in small and rural hospitals with basic labs without the need for referral centers. This will be a great advantage and an addition in the COVID era.

 Limitations of the study (some of the limitations are already discussed in the study).

1. The authors do not report the TB treatment nor considerations of the patients’ clinical outcome, so these tests are evaluated only in their performances in the diagnosis of TB (compared with the gold standard of culture), not considering the efficacy of treatment.
2. In the file “clinical data of the patients”, data on HIV treatment are very limited (see questions).
3. As at least two different types of LF-LAM tests are commercially available and have different performances, the author should explain the choice (LAM Alere Ag test) in the methods section and discuss in depth the performances of the different tests commercially available.
4. Intrinsic limitations of the sensitivity of LF-LAM were described in a recent paper of Tlali *et al. *(2020) ^1^ - the authors should discuss this paper.
5. POCUS examinations were performed by a single, experienced operator. A second blinded reviewer of the clips and/or sonographer would have added value to the results, considering that the US is a repeatable test.
6. The difference between the “individual” and “independent” aspect of the US is based on week evidence and considering that the result of this differentiation is that the focal splenic lesions and pleuric effusions, I’d delete it. Or, the author should just propose it separately and say that it needs further evaluation.

Questions and suggestions:

 HAART is a relevant issue in these patients. In the database, a considerable part of patients results on HAART therapy, so it is unclear why these patients were so deeply immunosuppressed; failure of the therapy? No compliance? The authors report that the patients are often confused at admission. This is true. However, the failure of the HAART therapy should both have had a role or be a consequence of TB infection: a brief comment of this aspect in the discussion should be appreciated. Also, I’ve noticed that no data on the VL are available, probably the test was not done. Please add it.

 Surprisingly, in the cohort, the different levels of CD4 considered by stratifying the patients in different groups with CD4 > of 200 or less. (<100 cells/mm3, 100–200 cells/mm3, >200 cells/mm3) does not have any impact. Probably, you should have considered another group with a higher level of CD4 and a working active therapy in the stratification (if that kind of patient were included in the cohort). If the CD4 doesn’t matter, also the HIV status could have been not so relevant as presumed in the performances of LF-LAM. You should add some considerations on these aspects.

 The neurological presentation was considered an exclusion criterion (Figure 1), but, in Table 1, 13 patients result to have a CRF sample. Please explain why these patients did a Lumbar Puncture, it should be interesting.

 In Table 3, I’d link with * the 63 patients with clinical diagnoses of tuberculosis of Table 3 with Table 4.

 Surprisingly, no one patient was diagnosed as ARL (AIDS-related lymphoma) that is the principal differential diagnosis (both clinical and the US) in TB-HIV patients: I'll speculate of some bias of selections (is it possible that patients with suspected lymphoma are preselected for referral centers? Sometimes it happens in African settings).

 Only 1 case of NTM was diagnosed in this cohort: it is interesting because in the work of Nel *et al*. (2017) ^2^ a considerable rate of false-positive LF-LAM test was found, and the cohort includes a high number of patients with a low CD4 count.

 In Table 4, some of the “Reason for diagnosis of tuberculosis without microbiological confirmation”, as “Suggestive formal abdominal ultrasound is done in radiology department” are really unclear, data on the true outcome of patients if available should be of particular interest.

 I partially disagree with the final consideration "The contribution of PoCUS in the context of urinary LF-LAM and chest X-ray availability was limited, due to the low positive yield, the need for further chest x-ray interpretation and the high false-negative rate...." because if you consider that LAM has good sensitivity but lower specificity, PoCUS whose specificity is very high could be the perfect tool. Please add the consideration to the conclusions. 

**Final comment**: it’s common thinking that “we have CXR, GeneXpert, and QGT, and we don’t need further tests”. Theoretically, it seems difficult to support the need for further tests for TB. But, in “real life”, there is a considerable “grey area” of challenging cases, particularly in patients with extrapulmonary TB, needing expensive second-level tests, like CT scan, Pet CT, laparoscopy with biopsy, staining and pathologists, all not available in LRS were most patients with TB live. The alternative is to start with an “empirical treatment” without any scientific evidence of TB except the WHO symptoms. But this is a rough approach in the MDR-TB era.

 The authors present a scenario, with a sidereal prevalence of TB (47%) and tremendously bad treatment of HIV. This peculiar situation might have influenced the results: other studies should have been carried in different settings, including a limited resource setting with a lower prevalence of HIV (and TB); a database including treatment and outcomes of the patients will add a lot. I suggest adding these considerations to the conclusions. To my knowledge, urinary LAM is increasingly used in LRS hospitals, but the test has some limitations and probably should be improved. However, probably there isn't a perfect test in TB and the combination of different tests is the better choice at the present moment. 

 I apologize to the editor and authors but my knowledge in statistics is limited, so my review is incomplete and only focused on the clinical aspect of the work. I think a mathematics or a statistics expert should be included as a reviewer of this manuscript

 Thank you for asking me to review this manuscript. I read it with high interest, and I found it accurate, written with conviction, and powerfully argued.

Is the work clearly and accurately presented and does it cite the current literature?

Yes

If applicable, is the statistical analysis and its interpretation appropriate?

I cannot comment. A qualified statistician is required.

Are all the source data underlying the results available to ensure full reproducibility?

Partly

Is the study design appropriate and is the work technically sound?

Yes

Are the conclusions drawn adequately supported by the results?

Partly

Are sufficient details of methods and analysis provided to allow replication by others?

Partly

Reviewer Expertise:

I'm a full-time clinician in Infectious Diseases and Tropical Medicine in San Bortolo Hospital, Vicenza, Italy. I started using ultrasound, POCUS and the interventional US in the setting of infective patients included HIV/TB patients in 1995 and exported my experience in remote settings with very constrained resources.

I confirm that I have read this submission and believe that I have an appropriate level of expertise to confirm that it is of an acceptable scientific standard, however I have significant reservations, as outlined above.

## References

[1] : Sensitivity of the lateral flow urine lipoarabinomannan assay in ambulant adults with advanced HIV disease: data from the TB Fast Track study. *Trans R Soc Trop Med Hyg* .2020; 10.1093/trstmh/traa018 10.1093/trstmh/traa018 32313941PMC7405171

[2] : Does Disseminated Nontuberculous Mycobacterial Disease Cause False-Positive Determine TB-LAM Lateral Flow Assay Results? A Retrospective Review. *Clinical Infectious Diseases* .2017;65(7) : 10.1093/cid/cix513 1226-1228 10.1093/cid/cix513 28575238

# Response

**Published:** 2022-05-06

**Authors:** Daniel van Hoving

**Affiliations:** University of Cape Town, South Africa

**Thank you to the time and effort to review the manuscript. Please find below our responses (in *bold italics*) to comments raised.**

 This manuscript is aimed to describe the use of LF-LAM in an acute care setting and identified PoCUS features independently associated with HIV-associated tuberculosis. Then, the study proposes a decisional algorithm for the diagnosis of TB in HIV patients, including point-of-care tests, as a result of a regression tree algorithm (CART).

 This algorithm combines the only two tests really available at the patient bedside and having the true characteristics of point of care test: LF-LAM and PoCUS, and the “classic” tests, CXR and GeneXpert PCR plus the culture of the sputum or pus: the two latter tests are now considered as the gold standard.

 The scenario is peculiar: the patients accessing this hospital have a high prevalence of HIV and are very deeply immunosuppressed, with very low CD4 count (never treated before for both conditions, or only treated for HIV).

**General comments**: Portable CXR and GeneXpert, reported in the text as “near point-of care-tests” really improved the opportunity of TB diagnosis in the last decades and now are the standard-of-diagnostic tools in many hospitals in many different resource settings. However, the clinical impact of these technologies on the TB epidemic and mortality was not so impressive, particularly if we consider patients living with HIV.

 The point-of-care tests like LF-LAM and PoCUS are available also in resource-limited settings (RLS) with affordable costs, can be performed in small and rural hospitals with basic labs without the need for referral centers. This will be a great advantage and an addition in the COVID era.

 Limitations of the study (some of the limitations are already discussed in the study).

1. The authors do not report the TB treatment nor considerations of the patients’ clinical outcome, so these tests are evaluated only in their performances in the diagnosis of TB (compared with the gold standard of culture), not considering the efficacy of treatment.

***This is correct. Patients that were empirically started on anti-tuberculous treatment were not included as cases. These participants were also not followed to determine whether they improved clinically. We did acknowledge this as a limitation.***

1. In the file “clinical data of the patients”, data on HIV treatment are very limited (see questions).

***Please see comment below***

1. As at least two different types of LF-LAM tests are commercially available and have different performances, the author should explain the choice (LAM Alere Ag test) in the methods section and discuss in depth the performances of the different tests commercially available.

***We only had access to one LF-LAM test (LAM Alere Ag test) when the study was initiated. We’ve explained the choice in the methods section as suggested. We’ve also added the estimated sensitivity and specificity of the FujiLAM test in the discussion.***

1. Intrinsic limitations of the sensitivity of LF-LAM were described in a recent paper of Tlali *et al. *(2020) ^1^ - the authors should discuss this paper.

***Thanks for the suggestion. The manuscript from Tlali et al evaluates the use of LF-LAM in ambulatory patients only and excluded patients too sick to be managed in ambulant care. The setting and population differ substantially to our study where participants were mostly acutely ill. The TB prevalence also differ markedly, 7% versus 42%. We therefor did not include the study in the discussion section.***

1. POCUS examinations were performed by a single, experienced operator. A second blinded reviewer of the clips and/or sonographer would have added value to the results, considering that the US is a repeatable test.

***We agree that a second blinded review of both the POCUS examinations and the chest x-rays would have added value to the results. This is acknowledged in the limitation section.***

 The difference between the “individual” and “independent” aspect of the US is based on week evidence and considering that the result of this differentiation is that the focal splenic lesions and pleuric effusions, I’d delete it. Or, the author should just propose it separately and say that it needs further evaluation.

***The individual and independent PoCUS features were based on a single study. We’ve acknowledge in the limitation section that this needs further evaluation.***

 Questions and suggestions:

 HAART is a relevant issue in these patients. In the database, a considerable part of patients results on HAART therapy, so it is unclear why these patients were so deeply immunosuppressed; failure of the therapy? No compliance? The authors report that the patients are often confused at admission. This is true. However, the failure of the HAART therapy should both have had a role or be a consequence of TB infection: a brief comment of this aspect in the discussion should be appreciated. Also, I’ve noticed that no data on the VL are available, probably the test was not done. Please add it.

***Overall 47% of participants were taking anti-retroviral therapy. We did not explore reasons for why participants were still deeply immune-supressed despite being on treatment. The viral load was unfortunately not done in participants.***

 Surprisingly, in the cohort, the different levels of CD4 considered by stratifying the patients in different groups with CD4 > of 200 or less. (<100 cells/mm3, 100–200 cells/mm3, >200 cells/mm3) does not have any impact. Probably, you should have considered another group with a higher level of CD4 and a working active therapy in the stratification (if that kind of patient were included in the cohort). If the CD4 doesn’t matter, also the HIV status could have been not so relevant as presumed in the performances of LF-LAM. You should add some considerations on these aspects.

***We unfortunately did not include a 4th group representing higher CD4 cell counts. We did comment on the use of urinary LF-LAM in participants with positive WHO screening symptoms regardless of CD4 cell count. This is an area that need further exploration.***

 The neurological presentation was considered an exclusion criterion (Figure 1), but, in Table 1, 13 patients result to have a CRF sample. Please explain why these patients did a Lumbar Puncture, it should be interesting.

***Only patients with a main neurological presentation were excluded. All laboratory tests performed by the hospital clinicians (non-research group) were evaluated and included if the tests related to tuberculosis. There is a very low threshold at the hospital to do a lumbar puncture as part of the evaluation of immunodeficient patients and hence the reason for including 13 CSF samples.***

 In Table 3, I’d link with * the 63 patients with clinical diagnoses of tuberculosis of Table 3 with Table 4.

***We have added a footnote to Table 3 linking the 63 patients to Table 4.***

 Surprisingly, no one patient was diagnosed as ARL (AIDS-related lymphoma) that is the principal differential diagnosis (both clinical and the US) in TB-HIV patients: I'll speculate of some bias of selections (is it possible that patients with suspected lymphoma are preselected for referral centers? Sometimes it happens in African settings).

***We are not aware that patients with suspected lymphoma are per-selected for referral centers at the study hospital. We can only speculate on why AIDS-related lymphoma was not diagnosed.***

 Only 1 case of NTM was diagnosed in this cohort: it is interesting because in the work of Nel *et al*. (2017) ^2^ a considerable rate of false-positive LF-LAM test was found, and the cohort includes a high number of patients with a low CD4 count.

***We can only speculate on why non-tuberculous mycobacterial infection was only cultured in one patient.***

 In Table 4, some of the “Reason for diagnosis of tuberculosis without microbiological confirmation”, as “Suggestive formal abdominal ultrasound is done in radiology department” are really unclear, data on the true outcome of patients if available should be of particular interest.

***We unfortunately did not follow patients up to see whether their clinical condition actually improves after the empiric initiation of anti-tuberculous treatment. We purposefully used the term ‘suggestive’ as there is no robust evidence on this (hence a motivation for the current study). The clinical diagnosis of tuberculosis also differ between attending physicians as some will only use certain ultrasound features (e.g. hypo echoic splenic lesions), where as others want to see more than one ultrasound feature (e.g. pericardial effusion and splenic lesions).***

 I partially disagree with the final consideration "The contribution of PoCUS in the context of urinary LF-LAM and chest X-ray availability was limited, due to the low positive yield, the need for further chest x-ray interpretation and the high false-negative rate...." because if you consider that LAM has good sensitivity but lower specificity, PoCUS whose specificity is very high could be the perfect tool. Please add the consideration to the conclusions. 

***We have added that the the role of POCUS as a rule-in test to diagnose HIV-associated tuberculosis needs to be further investigated***

**Final comment**: it’s common thinking that “we have CXR, GeneXpert, and QGT, and we don’t need further tests”. Theoretically, it seems difficult to support the need for further tests for TB. But, in “real life”, there is a considerable “grey area” of challenging cases, particularly in patients with extrapulmonary TB, needing expensive second-level tests, like CT scan, Pet CT, laparoscopy with biopsy, staining and pathologists, all not available in LRS were most patients with TB live. The alternative is to start with an “empirical treatment” without any scientific evidence of TB except the WHO symptoms. But this is a rough approach in the MDR-TB era.

 The authors present a scenario, with a sidereal prevalence of TB (47%) and tremendously bad treatment of HIV. This peculiar situation might have influenced the results: other studies should have been carried in different settings, including a limited resource setting with a lower prevalence of HIV (and TB); a database including treatment and outcomes of the patients will add a lot. I suggest adding these considerations to the conclusions. To my knowledge, urinary LAM is increasingly used in LRS hospitals, but the test has some limitations and probably should be improved. However, probably there isn't a perfect test in TB and the combination of different tests is the better choice at the present moment. 

 I apologize to the editor and authors but my knowledge in statistics is limited, so my review is incomplete and only focused on the clinical aspect of the work. I think a mathematics or a statistics expert should be included as a reviewer of this manuscript

 Thank you for asking me to review this manuscript. I read it with high interest, and I found it accurate, written with conviction, and powerfully argued.

# Peer review: Reviewer response for version 1

**DOI:** 10.21956/wellcomeopenres.17356.r38642

**Published:** 2020-06-01

**Authors:** Jim Connoly

**Affiliations:** 1 Department of Emergency Medicine, Newcastle upon Tyne Hospitals NHS Foundation Trust, Newcastle upon Tyne, UK

This is an extremely well written article of significant importance for the practice of EM in areas of the world with high endemic levels of Infectious diseases, specifically HIV and TB.

 It has been well powered, designed and conducted as a study and the presentation of results has been done in such a way as to recognises the limitations present in some low income systems that would affect the decision making tree, specifically by designing a number of trees with different starting points e.g. poor access to radiology. 

 In this regard FASH/PoCUS has been thought to have an increased role, specifically due to it’s portability and access at the bedside/out of hours and this work has sought to try and establish it’s role in any decision making tree. Similarly it has assessed the role of true point of care testing

**1 Manuscript**

 1.1 The research is well set out and easy to follow. The tables and figures are appropriately labelled and detailed.

 1.2 Editorially when printing it may be that the tables are moved around a little to be closer to where they are referenced.

**2 Study Design**

 2.1 The study design is appropriate for investigation of the subject and formulation of a decision tree.

 2.2 The study population as indicated is one of a high prevalence and so appropriate to study though, as the authors clearly indicate the applicability to areas of lower prevalence is more difficult to predict.

 2.3 Recruitment was good with respect to exclusion criteria some are obvious, others less obvious with no reason given as to exclusion of gynae/obstetric cases and CNS cases .There was a reasonable large amount excluded (132 in total) and some exclusions would prefer male over female inclusion (i.e. gynae/ obstetric though these appear small in number).

 I note however the male study population was only 40%. With respect to the exclusion criteria a clearer statement of any differences with the study group would be of value.

**3 Results**

 3.1 The use of phraseology “PoCUS independent and PoCUS individual features” is slightly unclear and could be clarified.

 3.2 Table 4 details the cases diagnosed with TB without microbiological confirmation - a total of 63 compared with a total of 172 microbiologically proven cases. This group would equate to 25% of patients finally treated for TB.

 I assume reading the paper these 25% were not included in the final statistics as the abstract clearly states the gold standard that the variables were looked at with respect to **microbiologically confirmed TB.**

 This data raises a few points of interest. I note in Table 4 almost 30% of the group diagnosed without microbiological proof were considered to have TB based on formal US findings. With my PoCUS interest it would be of great interest if the authors were able to review any reasons for differences in the US findings between the expert and PoCUS findings - this may be of interest for future improving of accuracy of any PoCUS algorithm.

 These 25% are also of potential interest when considering the results (see 4.2) .

**4 Discussion**

 4.1 This covers all the valid points/shortcomings of the study and gives clear evidenced recommendations. Although the authors comment that a weakness of the study was that it was performed under routine conditions in the emergency department, this in my view makes the research more applicable.

 4.2 Table 4 shows a number of patients in whom TB was diagnosed in the absence of microbiology which is over 25% of the final cases.

 The main scientific thrust of the paper has to be on confirmed cases as a scientific gold standard.

 However I do wonder in the final discussion if it is worth subjecting a few of the main decision trees and some of the univariable factors in Table 6 to data analysis which includes these cases to see how they might perform in a “real world” setting. This would obviously not be the main thrust of the scientific process but would be of great interest. Therein specifically, in light of the 30% being US findings I would be especially interested to see how PoCUS performed including that cohort of those eventually treated and whether it affects the number needed to scan?

 In regard to the performance of PoCUS, specifically 39 being needed to opt in one case – is it worth highlighting that this is however likely to increase in low prevalence and decrease in higher prevalence areas?

Is the work clearly and accurately presented and does it cite the current literature?

Yes

If applicable, is the statistical analysis and its interpretation appropriate?

Yes

Are all the source data underlying the results available to ensure full reproducibility?

Yes

Is the study design appropriate and is the work technically sound?

Yes

Are the conclusions drawn adequately supported by the results?

Yes

Are sufficient details of methods and analysis provided to allow replication by others?

Yes

Reviewer Expertise:

Point of Care Ultrasound.

I confirm that I have read this submission and believe that I have an appropriate level of expertise to confirm that it is of an acceptable scientific standard.

# Response

**Published:** 2022-05-06

**Authors:** Daniel van Hoving

**Affiliations:** University of Cape Town, South Africa

**Thank you to the time and effort to review the manuscript. Please find below our responses (in *bold italics*) to comments raised.**

 This is an extremely well written article of significant importance for the practice of EM in areas of the world with high endemic levels of Infectious diseases, specifically HIV and TB.

 It has been well powered, designed and conducted as a study and the presentation of results has been done in such a way as to recognises the limitations present in some low income systems that would affect the decision making tree, specifically by designing a number of trees with different starting points e.g. poor access to radiology. 

 In this regard FASH/PoCUS has been thought to have an increased role, specifically due to it’s portability and access at the bedside/out of hours and this work has sought to try and establish it’s role in any decision making tree. Similarly it has assessed the role of true point of care testing

**1 Manuscript**

 1.1 The research is well set out and easy to follow. The tables and figures are appropriately labelled and detailed.

 1.2 Editorially when printing it may be that the tables are moved around a little to be closer to where they are referenced.

**2 Study Design**

 2.1 The study design is appropriate for investigation of the subject and formulation of a decision tree.

 2.2 The study population as indicated is one of a high prevalence and so appropriate to study though, as the authors clearly indicate the applicability to areas of lower prevalence is more difficult to predict.

 2.3 Recruitment was good with respect to exclusion criteria some are obvious, others less obvious with no reason given as to exclusion of gynae/obstetric cases and CNS cases .There was a reasonable large amount excluded (132 in total) and some exclusions would prefer male over female inclusion (i.e. gynae/ obstetric though these appear small in number).

***All gynae/obstetric cases and CNS cases were not excluded, only patients with a main clinical presentation of meningitis syndrome or new focal neurology; gynaecological or psychiatric presentation.***

 I note however the male study population was only 40%. With respect to the exclusion criteria a clearer statement of any differences with the study group would be of value.

***Many HIV/TB studies have a female dominant sample population. We think this relates to cultural issues, where it is often seen as a weakness for young males to attend healthcare facilities.***

**3 Results**

 3.1 The use of phraseology “PoCUS independent and PoCUS individual features” is slightly unclear and could be clarified.

***Thanks for pointing this out. Independent POCUS features are POCUS features that were independently associated with tuberculosis and were determined by multivariable logistical regression. In this cohort, the independent POCUS features were pericardial effusion of any size, ascites, and intra-abdominal lymphadenopathy of any size. Individual POCUS features were any POCUS feature that showed an association with microbiologically confirmed tuberculosis without taking other POCUS features in consideration (i.e. univariable analysis) The statistical significant PoCUS feature with the lowest positive threshold was included, where different thresholds for positivity was used (eg, intra-abdominal lymph node size). This is explained in detail in the referenced study (Ref nr 22; Van Hoving DJ, Kenge AP, Maartens G, et al.: Point-of-Care Ultrasound Predictors for the Diagnosis of Tuberculosis in HIV-Positive Patients Presenting to an Emergency Center. J Acquir Immune Defic Syndr. 2020;83(4):415–423. 31904699 10.1097/QAI.0000000000002279), however we’ve reworded the paragraph under statistical analysis to better clarify the difference between the two types of POCUS features.***

 3.2 Table 4 details the cases diagnosed with TB without microbiological confirmation - a total of 63 compared with a total of 172 microbiologically proven cases. This group would equate to 25% of patients finally treated for TB.

 I assume reading the paper these 25% were not included in the final statistics as the abstract clearly states the gold standard that the variables were looked at with respect to **microbiologically confirmed TB.**

***This is correct. Only participants with microbiologically confirmation were included in the 172 cases***

 This data raises a few points of interest. I note in Table 4 almost 30% of the group diagnosed without microbiological proof were considered to have TB based on formal US findings. With my PoCUS interest it would be of great interest if the authors were able to review any reasons for differences in the US findings between the expert and PoCUS findings - this may be of interest for future improving of accuracy of any PoCUS algorithm.

***We did not compare PoCUS with radiology-performed ultrasounds. The 30% referred to, relate to participants without microbiological proof and it does not reflect any difference with POCUS findings. We will consider to determine the correlation between radiology-performed ultrasounds and POCUS findings as it would be of interest.***

 These 25% are also of potential interest when considering the results (see 4.2) .

***We did not include participants with ‘clinically-diagnosed tuberculosis’ as cases. Although initiating patients on empiric anti-tuberculous treatment is an accepted method in highly prevalent areas, we felt it would muddle the results as one never truly knows if the patient had tuberculosis or another disease susceptible to anti-tuberculous treatment.***

**4 Discussion**

 4.1 This covers all the valid points/shortcomings of the study and gives clear evidenced recommendations. Although the authors comment that a weakness of the study was that it was performed under routine conditions in the emergency department, this in my view makes the research more applicable.

 4.2 Table 4 shows a number of patients in whom TB was diagnosed in the absence of microbiology which is over 25% of the final cases.

 The main scientific thrust of the paper has to be on confirmed cases as a scientific gold standard.

 However I do wonder in the final discussion if it is worth subjecting a few of the main decision trees and some of the univariable factors in Table 6 to data analysis which includes these cases to see how they might perform in a “real world” setting. This would obviously not be the main thrust of the scientific process but would be of great interest. Therein specifically, in light of the 30% being US findings I would be especially interested to see how PoCUS performed including that cohort of those eventually treated and whether it affects the number needed to scan?

***Thanks for the comment. It would be interesting to see how POCUS performed when clinical cases were included. However, we did not follow up patients to determine whether they actually improved on anti-tuberculous treatment and this will severely limit the interpretation of such results.***

 In regard to the performance of PoCUS, specifically 39 being needed to opt in one case – is it worth highlighting that this is however likely to increase in low prevalence and decrease in higher prevalence areas?

***Thanks for the suggestion. We’ve incorporated it in the discussion section.***
